# Metavirome analysis of domestic sheep in Shaanxi, Gansu, and Ningxia, China

**DOI:** 10.3389/fvets.2024.1508617

**Published:** 2024-12-03

**Authors:** Sinong Zhang, Hui Gao, Gang Zhang, Min Fang, Yunyi Kong, Lingling Jiang, Qiang Liu, Pu Wang, Yanling Liu, Yong Li

**Affiliations:** ^1^College of Life Sciences, Ningxia University, Yinchuan, China; ^2^Key Laboratory of Conservation and Utilization of Biological Resources in Western China, Ministry of Education, Ningxia University, Yinchuan, China

**Keywords:** sheep, Shaanxi-Gansu-Ningxia region, viral metagenomics, RNA virus, DNA virus

## Abstract

Sheep play an important role in China’s agricultural development, but they are also potential hosts for many viruses, some of which have been identified as zoonotic pathogens, which may pose a serious threat to social public health and animal husbandry. Therefore, clarifying the characteristics of viruses in sheep will provide an important basis for the study of pathogenic ecology and viral evolution of viruses carried by sheep. We collected nasal and anal swabs from 688 sheep in 22 counties in Shaanxi, Gansu, and Ningxia, China, between January 2022 and July 2023, and utilized next-generation sequencing technology and bioinformatics approaches to identify the viruses in the samples. A total of 38 virus families carried by sheep were identified, including 12 ssRNA (+) virus families, 2 dsRNA virus families, 8 ssDNA (+) virus families, and 18 dsDNA virus families. Among them, Astroviridae, Coronaviridae, Picornaviridae, and Tobaniviridae in RNA virus families and Herpesviridae, Adenoviridae, and Circoviridae in DNA virus families are all viruses that are frequently detected in most ruminants. Alpha and beta diversity results showed that there was no difference in the overall richness and diversity of RNA and DNA viruses among the three provinces (*p* > 0.05). The evolutionary analysis demonstrated a tight link between the viral members carried by sheep and other ruminant viruses, implying that these viruses may spread across different species of ruminants. This study established a library of RNA and DNA viruses carried by sheep in the Shaanxi-Gansu-Ningxia region, providing an overview of the viruses present in this population. The findings offer valuable data for further research on virus evolution and monitoring in sheep.

## 1 Introduction

In taxonomy, sheep are classified as Artiodactyla, Ruminantinae, and Bovidae (1). They are widely disseminated over the world due to their importance in animal husbandry. China has an abundance of sheep breed resources, including 42 native breeds (2). The Shaanxi-Gansu-Ningxia region in China stands out due to its distinct geographical environment and extensive history of sheep breeding. According to statistics, the number of sheep in the region has reached 41.845 million,^1^ and the breeding methods are diverse, covering traditional free-range and large-scale breeding. However, this breeding model may also provide diverse environmental conditions for the spread and mutation of viruses (3–5). Existing research has primarily concentrated on the detection of particular common viruses, with no systematic investigation of the more complex and diverse viral communities and their properties that may occur in sheep. Therefore, it is of enormous significance to conduct an overall study on the viruses carried by sheep in the Shaanxi-Gansu-Ningxia region.

Metaviromics is a technical means of sequencing and analyzing all viral populations in the host or environment. Next Generation Sequencing (NGS) can give higher resolution and more complete genomic or transcriptome information for identifying viruses in biological samples (6). Social and public health have applied metaviromics to monitor the biosafety of potential pathogenic viruses (7). In recent years, metaviromics has revealed the diversity and complexity of a lot ruminant livestock viruses (8). The metaviromics analysis of ruminant feces and respiratory samples not only identified a wide range of known viruses like astroviruses (9), coronaviruses (10), and enteroviruses (11), but also discovered the new bovine rhinitis B virus and the newly proposed prevalence of ungulate bocavirus 6 (12). Simultaneously, it revealed a rich abundance of bacteriophages in the ruminant gastrointestinal tract (8, 13). These research are critical for us to monitor the status of viruses in ruminants, understand their evolution, and avoid future illness outbreaks.

Shaanxi, Gansu, and Ningxia are important animal husbandry regions in my country, but there is currently a lack of research on the virus-carrying status of livestock in the region, resulting in a lack of background data on the virus-carrying status of livestock here, and it is unclear whether the virus-carrying status poses a public safety risk. As a result, this study collected nasal and anal swab samples from 688 sheep in 22 counties across Shaanxi, Gansu, and Ningxia, and used metagenomics methodologies to determine the virus-carrying status of the sheep in the region.

## 2 Materials and methods

### 2.1 Sample collection and processing

**Figure 1:**
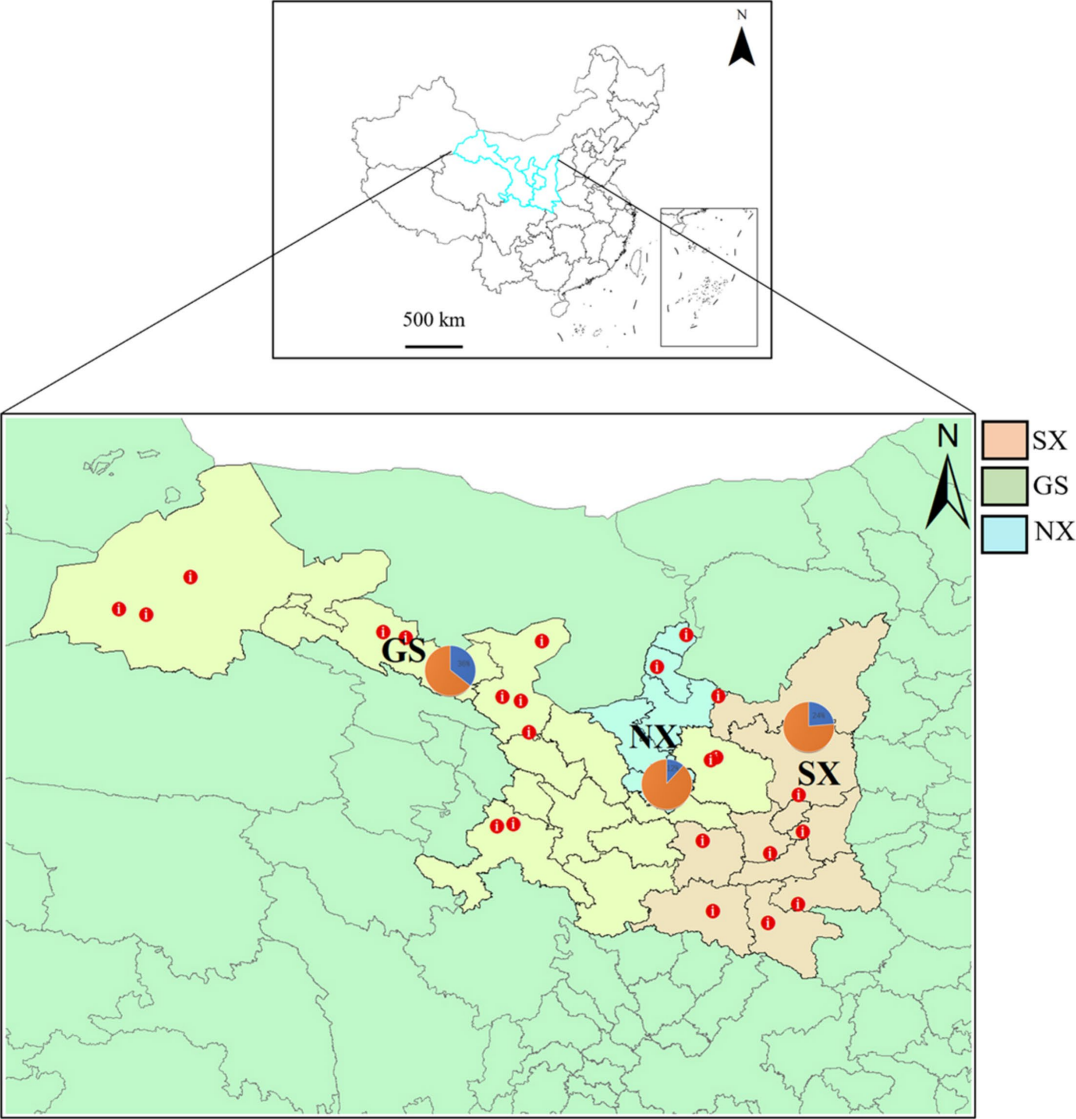
Distribution of sheep sampling sites in Shaanxi, Gansu, and Ningxia. The sampling sites are marked with red dots on the map (GS, Gansu Province; SX, Shaanxi Province; NX, Ningxia Hui Autonomous Region), and the pie chart shows the proportion of sample collection in each province.

We designed sampling points based on the sheep breeding conditions in Shaanxi Province, Gansu Province and Ningxia Hui Autonomous Region (Figure 1). A total of 688 sheep nasal and anal swab samples were collected from January 2022 to July 2023 (Table 1). Samples were collected by professional veterinary staff with the consent of the sheep owners, and all surveyed animals had no previous disease records. A disposable sterile swab was inserted into the nose and anus of the sheep and rotated 3 to 4 times for collection. The swab neck was then broken and placed in a sterile virus preservation solution, which was transported to the laboratory on dry ice and stored at −80°C. The consumables used in sample processing were all Ribozyme-free Brands, and all operations were performed in a fume hood. We processed the nasal and anal swab samples from each province according to the three provinces. After the samples were vortexed for 10 min, the supernatant was collected by centrifugation at 12,000 rpm for 10 min at 4°C. At the same time, in order to remove fungi and bacteria in the samples, we filtered the supernatant through 0.45 μm and 0.22 μm filters (Millipore) and then concentrated the virus in the supernatant using a 50KD Millipore ultrafiltration tube. Finally, the samples were divided into 3 RNA sample pools and 3 DNA sample pools according to the sampling provinces.

**Table 1:** Sample information in Shaanxi, Gansu, and Ningxia.

Region	Sampling time	Sampling sites	Number
Shaanxi province	July 2023	7	213
Gansu province	April 2023	12	383
Ningxia province	January 2022	3	92
Total		22	688

### 2.2 Metagenomic library construction and sequencing

In order to comprehensively analyze the distribution of RNA and DNA viruses in the samples, we constructed RNA and DNA sample pools from three regions for sequencing. We extracted RNA from RNA virus libraries using the Viral RNA Micro Kit (Qiagen Biotech) and removed host rRNA using the Hieff NGS MaxUp rRNA Removal Kit (Yisheng Biotech). Fragmentation buffer (Thermo Fisher Scientific) was added to the enriched RNA to break the RNA into small fragments. Then, the fragmented RNA was used as a template, 6 bp random primers were added for reverse transcription to synthesize the first strand of cDNA, and buffer, dNTPs, DNA polymerase I, and RNase H were added to synthesize the second strand of cDNA. After the synthesized double-stranded cDNA was purified, end-repaired, A-added, and ligated to the sequencing adapter, the second strand of cDNA containing U was degraded with the USER enzyme and PCR-enriched. Finally, we purified the PCR product using AMPure XP beads (Beckman Coulter) to obtain the final strand-specific library. The quality and concentration of RNA were then evaluated based on the Agilent 2,100 Bioanalyzer system (library effective concentration > 2 nM) to ensure the quality of the library. For the DNA virus library, we used an oral swab genomic DNA extraction kit (Tiangen Bio) to extract DNA, amplified the genome with the Illumina GenomiPhi V2 DNA Amplification Kit (Cytiva), and then purified it with a large amount of the DNA product purification kit (Tiangen Bio). Finally, we sequenced all libraries using Beijing Novogene Technology Co., Ltd.’s Illumina HiSeq XTen platform to produce 300 bp paired-end reads. During the sequencing process, all libraries were treated with NFW (nuclease-free water) as a blank control.

### 2.3 Analysis of viral diversity and abundance

To find out how common different viral families and species were in the library, we first used the Trimmomatic program to check the quality of the sequence data and get rid of low-quality sequences, reads that were less than 36 bp, and Illumina-specific sequencing adapter sequences (14). The resulting reads (non-rRNA) were then further assembled *de novo* using the Trinity program under default settings (15). Bowtie2 (16) was then used to map the trimmed reads to the host reference genome (*Ovis aries*: ARS1) under default parameters while using the Illumina quality control template (PhiX174). After retaining the unmapped reads, Velvet Optimiser was further used to complete the *de novo* assembly and generate continuous sequences (contigs) (17). The resulting contigs were compared to non-redundant nucleotide (NT) and protein (NR) databases using Blastn and Blastx, respectively. We set the *E*-value threshold to <1 × 10^−5^ to eliminate false positives (18), and unclassified viral reads were annotated separately and not included in the diversity analysis. After BLAST analysis, we then evaluated the diversity and abundance of viruses at the family and genus levels in the libraries based on Alpha and Beta diversity and used the Kruskal-Wallis rank sum test to compare and detect differences between libraries. We performed PCoA principal coordinate analysis using the vegan and ggplot2 software packages to characterize the structural distribution of viral information at the family and genus levels in each library.

### 2.4 Phylogenetic analysis

To confirm the phylogenetic relationships of the viruses found in this study, representative reference virus sequences were retrieved from the GenBank database based on the predicted nucleotide sequences and the best matching results of Blastn and Blastx and aligned using ClustalW provided in MEGA 7.0 under default settings (19). The aligned sequences were trimmed to match the gene sequences required for the study, and the sequences were compared for phylogenetic analysis using the maximum likelihood method in MEGA version 7.0. Each analysis was performed with 1,000 repeated bootstrap analyses, and nodes with bootstrap values greater than 70% were retained (20). FigTree v.1.4.4^2^ was then used to annotate and modify the phylogenetic tree.

### 2.5 Data availability

All raw sequence reads obtained in this experiment are available in the NCBI Short Read Archive under BioProject with accession number PRJNA1155242, and all viral genome sequences have been submitted in GenBank with accession numbers PQ573796 to PQ573826 (sequences are supplied in Appendix 1).

## 3 Results

### 3.1 Overview of metagenomic sequencing data

There were 715,512,56 Clean Reads in Shaanxi Province SXY-RNA, of which 331,41 were viruses, accounting for 0.05%; there were 717,322,50 Clean Reads in Gansu Province GSY-RNA, of which 284,68 were viruses, accounting for 0.04%; there were 444,532,36 Clean Reads in Ningxia Hui Autonomous Region NXY-RNA, of which 317,12 were viruses, accounting for 0.07% (Table 2).

**Table 2:** Overview of sequencing libraries of different macroviromes.

Group	RNA concn (ng/μL)	No. of reads	No. of clean reads	No. of contigs	Viral read abundance (%)
SX-RNA	491.00	71,551,292	71,551,256	89,892	0.05
GS-RNA	354.00	71,732,256	71,732,250	60,113	0.04
NX-RNA	220.9	44,598,390	44,453,236	88,024	0.07
SX-DNA	36.69	72,267,200	72,267,200	99,007	6.69
GS-DNA	54.43	69,729,938	69,729,938	19,256	0.56
NX-DNA	59.20	75,844,452	75,844,452	238,511	6.35

There were 72,267,200 Clean Reads in Shaanxi Province SXY-DNA, of which 4,834,676 were viruses, accounting for 6.69%; there were 69,729,938 Clean Reads in Gansu Province GSY-DNA, of which 390,488 were viruses, accounting for 0.56%; there were 75,844,452 Clean Reads in Ningxia Hui Autonomous Region NXY-DNA, of which 4,816,123 were viruses, accounting for 6.35% (Table 2).

### 3.2 Analysis of diversity of virus communities carried by sheep

**Figure 2:**
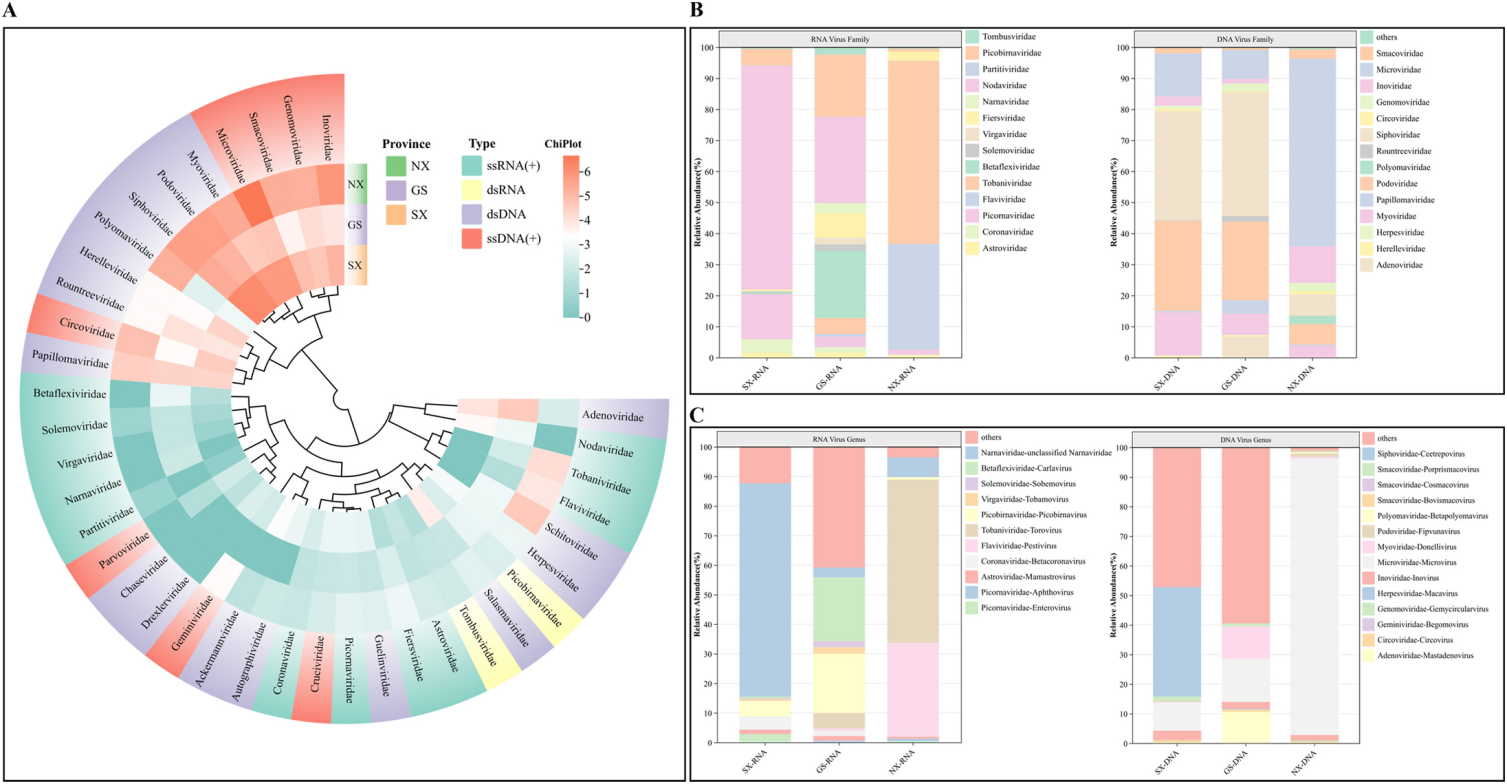
Classification analysis of sheep virus reads at the family and genus level. **(A)** Heat map showing the abundance of reads in different families of RNA/DNA viruses. The number of reads was log2 transformed; **(B)** The proportion of different families of RNA and DNA viruses. **(C)** The proportion of RNA and DNA viruses from different genera.

After BLAST alignment, the viral reads were finally annotated to 38 viral families. Among RNA viruses, Astroviridae, Coronaviridae, Picornaviridae, Flaviviridae, Tobaniviridae, Betaflexiviridae, Solemoviridae, Virgaviridae, Fiersviridae, Narnaviridae, Nodaviridae, and Partitiviridae have single-stranded positive-sense RNA [ssRNA(+)] genomes, and Picobirnaviridae and Tombusviridae have double-stranded RNA (dsRNA) genomes (Figure 2A). Among DNA viruses, viruses from the Adenoviridae, Herpesviridae, Papillomaviridae, Polyomaviridae, and Myoviridae families have double-stranded DNA (dsDNA) genomes; viruses from the Circoviridae, Geminiviridae, Genomoviridae, and Inoviridae families have single-stranded positive-sense DNA [ssDNA(+)] genomes (Figure 2A).

At the level of specific families and genera, Tobaniviridae-Torovirus is the virus with the highest proportion of all RNA viruses, accounting for as high as 46.76%, of which the proportion of the virus in Ningxia Hui Autonomous Region is 59.02%; Flaviviridae-Pestivirus accounts for 26.77% of all RNA viruses, and the proportion of the virus in Gansu Province is 0.83%, and the proportion in Ningxia Hui Autonomous Region is 34.02%; Astroviridae and Coronaviridae account for 0.85 and 0.96% of all RNA viruses, respectively. The virus has been found in samples from three provinces, and the two viruses are widely distributed; Nodaviridae accounts for 11.71% of all RNA viruses, the highest proportion of viruses in Shaanxi Province. In addition, plant viruses Betaflexiviridae, Solemoviridae, Tombusviridae, and Virgaviridae account for 2.99% of all RNA viruses. For DNA viruses, bacteriophages account for 94.51%, and animal viruses account for 5.49%. Among them, Genomoviridae-Gemycircularvirus, Polyomaviridae-Betapolyomavirus, Circoviridae-Circovirus, Adenoviridae-Mastadenovirus, and Herpesviridae-Macavirus accounted for 2.02, 1.52, 0.89, 0.39, and 0.02%, respectively (Figures 2B,C).

### 3.3 Analysis of viral community composition

**Figure 3:**
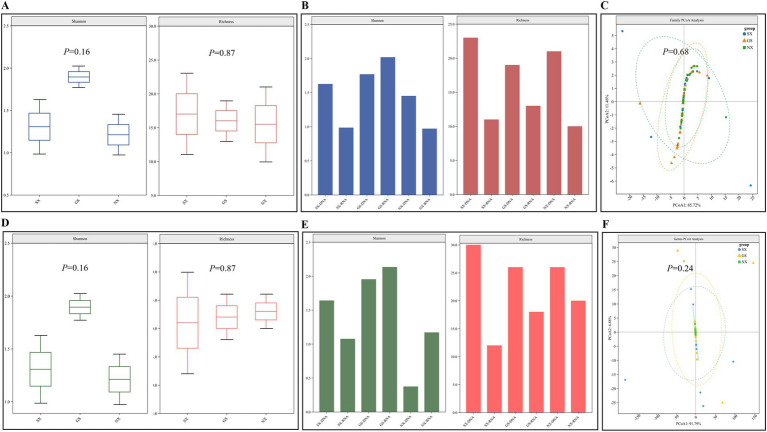
Analysis of the diversity of viruses carried by sheep. **(A,D)** *β* diversity analysis at the level of RNA/DNA virus family and genus; **(B,E)** Alpha diversity analysis at the level of RNA/DNA virus family; **(C,F)** Alpha diversity analysis at the level of RNA/DNA virus genus.

Alpha diversity can well characterize the diversity and richness of viruses in different sample pools. Richness can directly reflect the number of virus species in the sample pool, and the Shannon index reflects the degree of diversity in the sample pool. The larger the value, the higher the diversity of viruses in the sample pool. The results of Alpha diversity analysis of RNA and DNA virus libraries in samples from the three provinces of Shaanxi, Gansu, and Ningxia showed that there was no difference in the richness and diversity of species of RNA and DNA viruses at the family and genus levels in the samples of the three provinces (*p* > 0.05) (Figures 3A,D). However, it can also be found that the Alpha diversity of RNA and DNA viruses in Gansu Province is higher, while the Alpha diversity in Ningxia Hui Autonomous Region is lower (Figures 3B,E). *β*-diversity can reflect the differences in virus diversity between different sample pools. Among them, PCoA (principal coordinate analysis) can well reveal the similarities and differences of viruses between different sample pools. Our *β*-diversity analysis of RNA and DNA virus libraries in samples from the three provinces of Shaanxi, Gansu, and Ningxia showed that the viruses contained in each library had many similar components at the family and genus levels and shared multiple viruses (*p* > 0.05) (Figures 3C,F).

### 3.4 Genetic evolution analysis of sheep viruses

#### 3.4.1 Evolutionary analysis of RNA viruses

##### 3.4.1.1 Astroviridae

**Figure 4:**
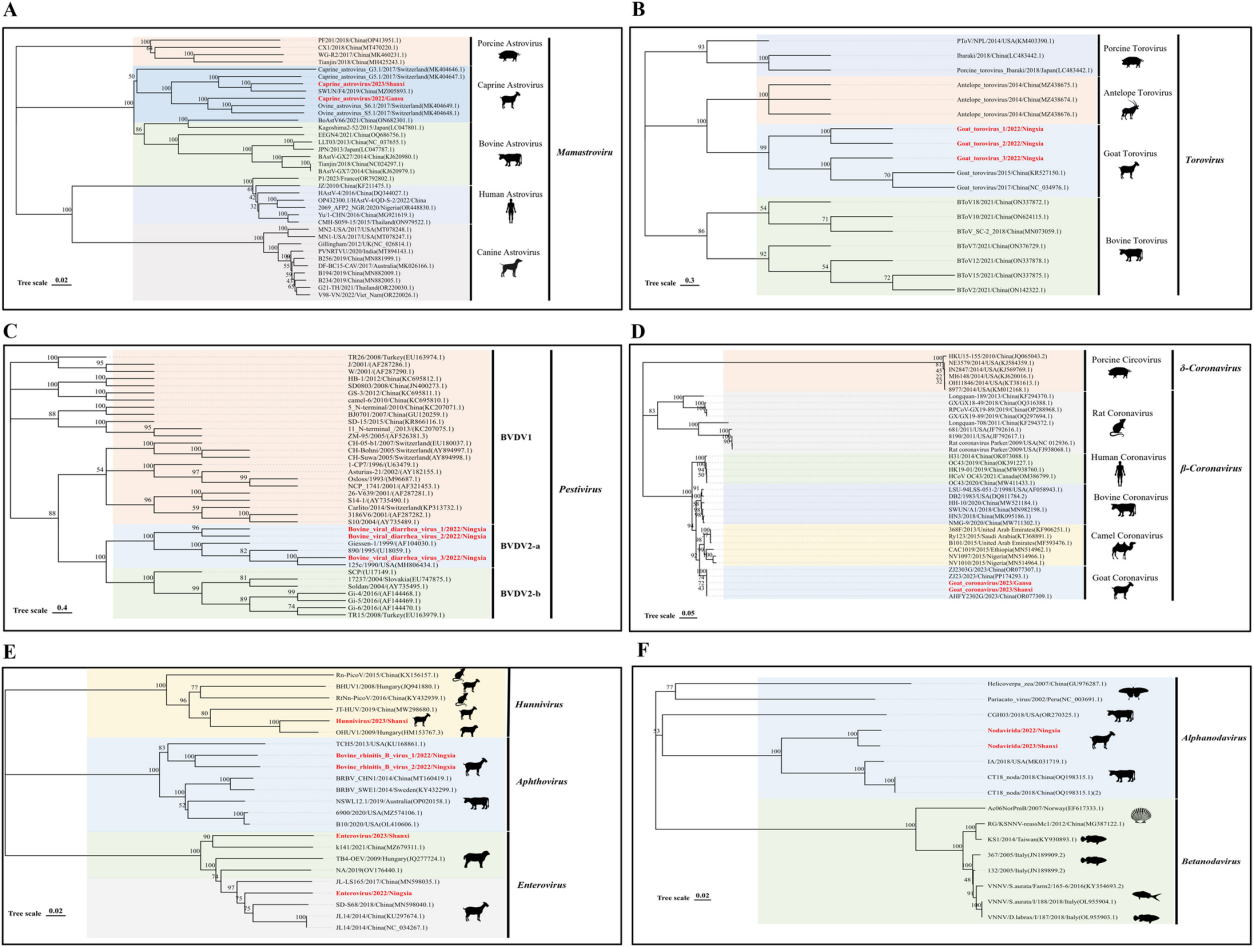
Genetic evolution analysis of RNA viruses. **(A)** Phylogenetic tree constructed based on the ORF1a nucleic acid sequence of Astroviridae; **(B)** N gene sequence of Tobaniviridae; **(C)** N-terminal protease nucleic acid sequence of Flaviviridae; **(D)** S gene sequence of Coronaviridae; **(E)** Partial gene sequence of Picornaviridae; **(F)** Representative viruses in the A gene family of Nodaviridae. The sequences of the strains in this study are shown in red bold font.

Astroviridae are widely found in mammals, and co-infection with this virus and other viruses can cause diarrheal diseases. We found the presence of astrovirus in the three RNA libraries in Shaanxi, Gansu, and Ningxia and assembled two ORF1a nucleic acid sequences of the virus (1064–1,515 bp), which had the highest similarity of 85–89% with the nucleic acid sequence uploaded by Switzerland in 2017 (GenBank No. MK404647.1); in phylogenetic relationships, they all belong to the *Mamastrovirus* genus, and all ovine astroviruses eventually clustered on the same branch (Figure 4A), indicating that the virus may form a new evolutionary branch in the sheep population.

##### 3.4.1.2 Tobaniviridae

Tobaniviridae was first discovered in 1979 on a farm in Breda, Iowa, USA (21). Currently, this virus family has been reported in various mammals, including human torovirus, porcine torovirus, and bovine torovirus, and has become an important intestinal pathogen in humans and animals. We assembled three nearly complete *N* gene nucleic acid sequences (492 bp) of Tobaniviridae in the Ningxia RNA library. These three sequences have 98–99% nucleotide identity with the sequence *Goat torovirus SZ* (GenBank No. KR527150.1) uploaded from China. Phylogenetic analysis results showed that all three sequences belong to the genus *torovirus* (Figure 4B).

##### 3.4.1.3 Flaviviridae

The pestivirus genus in Flaviviridae includes *pestivirus type A (BVDV-1)*, *pestivirus type B* (*BVDV-2*), and *pestivirus type H*, which can cause respiratory and digestive tract diseases in animals after infection. We found the presence of this virus in the Ningxia RNA library and performed sequence alignment and evolutionary analysis based on the *N-terminal protease* gene. The nucleic acid sequence alignment results showed that the three sequences were 99% similar to the sequence *125c* (GenBank No. MH806434.1) uploaded from the United States; in the phylogenetic relationship, the three sequences clustered on the DVDV2-a branch (Figure 4C). This type of virus was first found in sheep in my country, indicating that the BVDV2-a virus has been prevalent in sheep flocks in Ningxia, my country.

##### 3.4.1.4 Coronaviridae

Coronaviridae is an important pathogen that causes intestinal and respiratory diseases in humans and animals and has significant genomic and biological variability in different hosts (22). Among them, the spike glycoprotein (S) is an important protein for coronavirus to invade hosts and cause pathogenicity. At the same time, the mutation of this protein is also an important factor in the expansion of the host range of the virus (23). We assembled two *S* gene nucleic acid sequences (688–4,089 bp) of the virus in the Shaanxi and Gansu RNA libraries. These two sequences are 99% similar to the *AHFY2302G* (GenBank No. OR077309.1) nucleic acid sequence uploaded by China in 2023; the phylogenetic results show that they all belong to the *β-Coronavirus* genus and are clustered on the same branch as the sheep-derived coronavirus sequence uploaded by my country in 2023, suggesting that the virus may form an independent evolutionary branch in my country’s sheep population (Figure 4D).

##### 3.4.1.5 Picornaviridae

Picornaviridae is a relatively abundant virus family in mammals and is an important pathogen that causes respiratory and digestive tract abnormalities in mammals. We identified three virus genera in this family, namely *Hunnivirus*, *Aphthovirus*, and *Enterovirus*. We obtained a partial nucleic acid sequence of the *Hunnivirus* genus in the Shaanxi RNA library, which has a sequence identity of 91% with *OHUV1/2009/HUN* (GenBank No. HM153767.3) uploaded from Hungary and is located on the same evolutionary branch. The two sequences of the *Aphthovirus* have a similarity of 80 and 78% with *bovine rhinitis B virus 1* (GenBank No. NC_010354.1) and *USII/19* (GenBank No. KU159359.1) uploaded from the United States, respectively. Both sequences were collected from cattle, so the virus may have been transmitted from cattle to sheep. At the same time, the two enterovirus sequences we assembled belonged to the G serotype, and their similarity with the sequence *JL14* (GenBank No. KU297674.1) obtained from Chinese goats was 79–87% (Figure 4E).

##### 3.4.1.6 Nodaviridae

Nodaviridae were first discovered in Culex tritaeniorhynchus in Noda Village, Japan. Among them, the *Alphanodavirus* genus mainly infects insects and mammals, while the *Betanodavirus* genus infects invertebrates such as fish. In this study, we discovered the presence of Nodaviridae in sheep herds in Shaanxi and Ningxia for the first time and assembled two *A* gene nucleic acid sequences of Nodaviridae (950–1,653 bp). The results of nucleotide sequence alignment showed that they were 75% similar to the sequence *CT18 noda* (GenBank No. OQ198315.1) found in cattle in my country; the phylogenetic tree results showed that the two sequences were clustered on the same branch as the cattle source (Figure 4F).

#### 3.4.2 DNA virus evolution analysis

##### 3.4.2.1 Circoviridae

**Figure 5:**
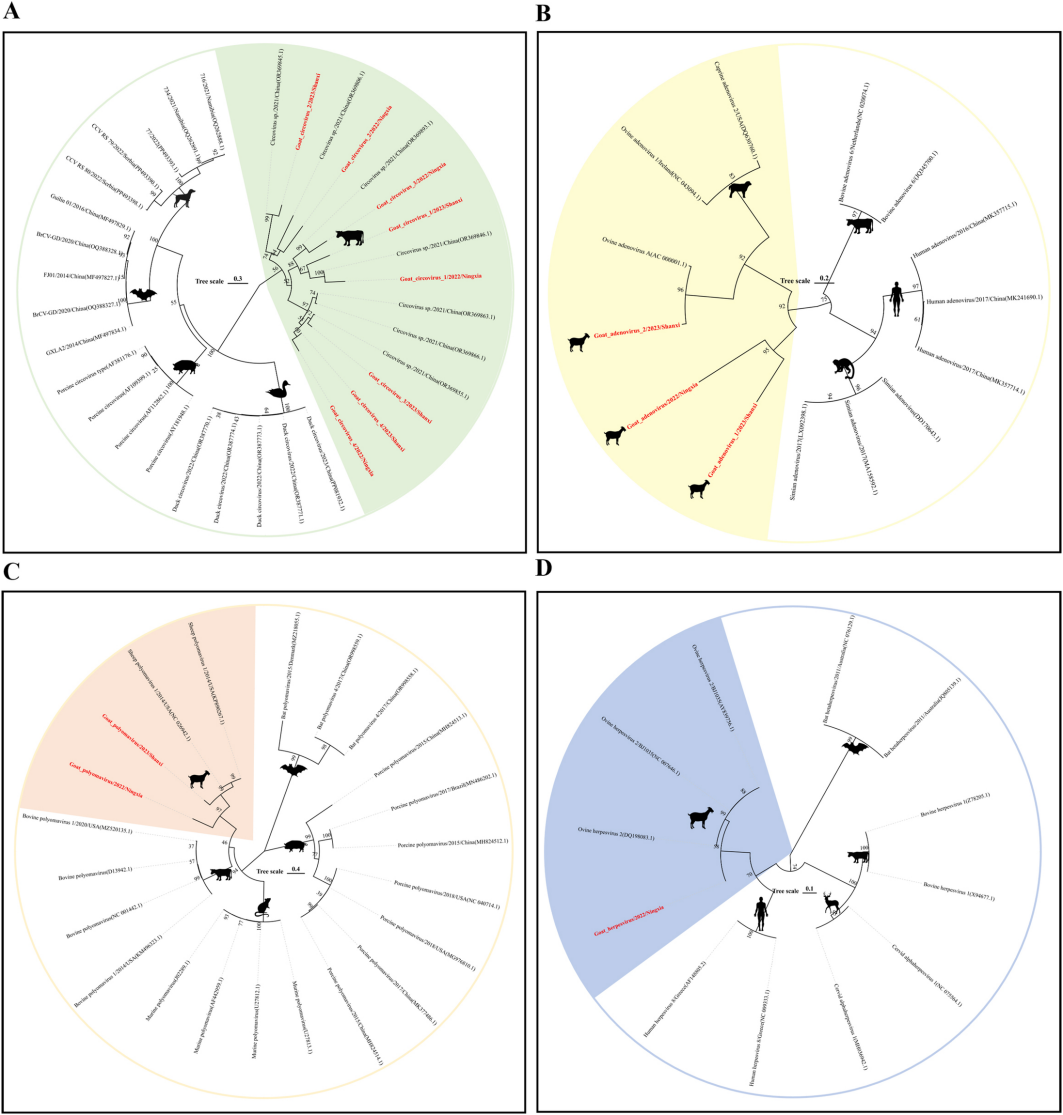
Genetic evolution analysis of DNA viruses. **(A)** Phylogenetic tree constructed based on the Rep gene sequence of Circoviridae; **(B)** Hexon gene family of Adenoviridae; **(C)** VP1 gene sequence of Polyomaviridae; **(D)** Dpol gene sequence of Herpesviridae. The sequences of the strains in this study are shown in red bold font.

Circovirus is a small, non-enveloped, single-stranded DNA virus that mainly encodes two proteins, replicase (Rep) and capsid protein (Rep). Mammals are its natural hosts, and birds and poultry are also hosts of the virus (24). In this study, we spliced six circovirus *Rep* nucleic acid sequences (691–1,023 bp) from DNA libraries in three provinces. They were 80–95% similar to the circovirus sequences reported in China; evolutionary analysis results showed that these six sequences all belonged to the genus Circovirus and were clustered on the same large branch as the bovine circovirus. Further analysis results showed that they were on different small branches, which revealed the rich genetic diversity of the virus in sheep (Figure 5A).

##### 3.4.2.2 Adenoviridae

Adenovirus is a type of non-enveloped, dodecahedral, double-stranded DNA virus. *Mastadenovirus* has only been isolated from mammals. The virus can cause respiratory and intestinal infections in mammals (25). We spliced three nearly complete *Hexon* gene nucleic acid sequences (1766–2098 bp) from the Shaanxi and Ningxia DNA libraries. When aligning the nucleic acid sequences, we mapped the three sequences to *ovine adenovirus A* (GenBank No. AC 000001.1). The results showed that the nucleic acid sequence similarity between them was more than 90%; phylogenetic analysis showed that they all belonged to the *Mastadenovirus*. This shows that the prevalence of ovine adenovirus A is more common in sheep herds in the Shaanxi-Gansu-Ningxia region of my country (Figure 5B).

##### 3.4.2.3 Polyomaviridae

Polyomaviridae is a small double-stranded DNA virus that can infect a variety of mammals, birds, and fish. In research, the virus is named because it can induce changes in infected host cells, and some viruses have tumorigenic activity in animals (26). In this study, we spliced two complete *VP1* gene nucleic acid sequences (1,185 bp) of the Polyomaviridae family from the Shaanxi and Ningxia DNA libraries. The sequence comparison results showed that the highest similarity with the nucleic acid sequence of *sheep polyomavirus 1* (GenBank No. NC 026942.1) uploaded from the United States was 80%. In terms of evolutionary development, they are both on the same evolutionary branch (Figure 5C), and the virus has not been classified at the genus level.

##### 3.4.2.4 Herpesviridae

Herpesvirus is a type of icosahedral double-stranded DNA virus, which can be divided into three subfamilies: *α*, *β*, and *γ*. It can infect birds, humans, and other mammals and cause respiratory diseases in mammals (27). We obtained a 992 bp Dpol nucleic acid sequence in the Ningxia DNA library. Sequence alignment results showed that the sequence had a 99% similarity with the nucleic acid sequence of *ovine gammaherpesvirus 2* (GenBank No. NC 007646.1) found in sheep in the United States in 2016. The results of phylogenetic analysis showed that the sequence was on the same evolutionary branch as ovine herpesvirus 2 and belonged to the *Gammaherpesvirinae* subfamily (Figure 5D).

## 4 Discussion

With the widespread application of metagenomics technology in agriculture, medicine, and environmental monitoring, the genetic information of many new viruses has been revealed, but there are still many viruses that need to be identified. According to some study, more than 40,000 distinct viruses are parasitic in mammals, greatly exceeding the number of viruses certified by the International Committee on Taxonomy of Viruses (ICTV) (28, 29). It is estimated that about 80% of the viruses known to infect humans can parasitize in other non-human “hosts,” including farm mammals, poultry, and wild animals. In this study, a large number of mammals were raised in the Shaanxi-Gansu-Ningxia region, among which the scale and number of sheep farming accounted for a particularly large proportion. For a long time, there have been reports of epidemics of sheep and other mammals in the region, and some diseases may be transmitted to humans, resulting in human infectious disorders. In view of this, this study collected nasal swab and anal swab samples from sheep in three provinces and used metagenomic technology to study the richness and evolutionary relationships of viruses carried by sheep to assess whether they pose a potential threat to human and animal health.

In our samples, the most common vertebrate virus representatives were found, but no new species or highly pathogenic viruses were reported. In addition, the viral communities showed generally similar characteristics in the region. The samples used in this study had a lot of Torovirus, which is a pathogen that can make people and animals sick with digestive problems (30). It made up 46.76% of all the RNA virus library reads. This result shows that the virus is ubiquitous in the samples of this study. The virus was first reported in calves with severe diarrhea in 1979. In 1984, the virus was detected in fecal samples of adults and children with gastroenteritis in the UK and France using electron microscopy (31). With the continuous deepening of research on the virus, the prevalence of Torovirus has been detected in domestic animals and wild animals in countries around the world, including China, France, Germany, the Netherlands, and South Africa (32, 33). Although Torovirus infection does not show serious symptoms in adult animals, studies have shown that frequent interactions and internal recombination between viruses can increase pathogenicity or unpredictable host adaptability (30). These findings highlight the importance of Torovirus as a zoonotic pathogen and the need for basic research on this virus.

At the same time, we found a substantial number of nucleic acid sequences from the Astroviridae, Coronaviridae, and Picornaviridae families in the RNA library. Previous studies have shown that the prevalence of these viruses can be detected in multiple samples from healthy or sick domestic ruminants (cattle, sheep, and camels) and wild ruminants (deer, antelope, and yaks) (34–36). In our study samples, the astrovirus strains were most closely related to the *goat astrovirus G5.1* from Switzerland (GenBank No. MK404647.1), which and other goat or sheep astrovirus strains in China belong to the genotype species of the *Mamastrovirus* genus that was previously identified as a goat-sheep AstV recombinant strain (“*goat astrovirus G5.1*”) (37, 38). These findings suggest that this type of astrovirus strain is widespread both at home and overseas.

In addition, bovine coronavirus has been confirmed to be an important cause of enteritis and lamb mortality in domestic sheep (39). The coronavirus strain in this study is most closely related to the Chinese bovine coronavirus *AHFY2302G* (GenBank No. OR077309.1), both belonging to the genus *Betacoronavirus* and species *Betacoronavirus-1*, which is currently considered to be a mutant of the bovine coronavirus that infects most ruminants (40). The high frequency of genetic changes in coronaviruses makes them zoonotic (41). Although bovine coronavirus has been found to be prevalent and spread in sheep, there are relatively few studies on sheep coronavirus, and its pathogenicity remains largely unknown. These viruses do not cause serious diseases when they infect the host alone, but they may interact with other viruses or microorganisms, thereby aggravating the severity of the host’s disease or even causing its death (42, 43). Simultaneously, genetic recombination between strains might result in the formation of novel variations, increasing virulence or cross-species risk. This has become a major topic in current social public health monitoring and research.

In the DNA virus library, the number of phage reads accounted for 94.51%, indicating that phages accounted for a very high proportion in this study. Ruminant gastrointestinal tract (GIT) microorganisms have been proven in studies to be essential bacterial communities capable of digesting lignocellulose and other plant foods and protecting animals from dangerous bacteria and infections (44, 45). Phages are an important component of the ruminant gastrointestinal tract (GIT) microbiome and play a vital role in forming microbial structure and function. They are ideal tools for inhibiting the growth of methanogenic archaea in the gastrointestinal tract (46, 47). In our study, the Microviridae, Siphoviridae, Myoviridae, and Podoviridae families were the main families, which is consistent with the phage catalogs of other ruminant gastrointestinal metagenomic studies (8, 48). At the same time, the majority of our samples came from farmers or small-scale free-range pastoral areas with a complicated breeding environment, which could explain the high proportion of bacteriophages.

In addition, common vertebrate virus families such as Circoviridae, Herpesviridae, and Adenoviridae were found in our DNA virus library, but no highly pathogenic viruses were identified. A large number of these viruses were found in the same metagenomic studies of yaks in the Qinghai-Tibet Plateau and in the analysis of the viral community of ruminants in the northwest plateau of China (13, 49). Studies have shown that circoviruses can infect both vertebrates (including mammals and birds) and some invertebrates (50). Currently, there are only a few reports on circoviruses in sheep. In our study, all circoviruses clustered on the same branch as bovine circoviruses, while Boros et al. (51) found that circoviruses in goats clustered together with circoviruses in humans and rhesus monkeys on the evolutionary branch, which indicates the genomic diversity of the virus in sheep and the possibility of cross-species transmission.

We also discovered other viruses that are closely related to plant viruses, such as RNA viruses from the Betaflexiviridae, Solemoviridae, Tombusviridae, and Virgaviridae families and DNA viruses from the Genomoviridae family. Previous research has also shown that some plant viruses occur in mammals (49, 52). This suggests that these viruses may have some kind of biological interaction with mammals. Therefore, further research on the interaction between plant viruses and mammals, the transmission mechanism, and the impact on the host will help us gain a deeper understanding of the diversity of viruses and their potential threats to biological health.

Despite conducting a comprehensive study on the viruses carried by sheep in the Shaanxi-Gansu-Ningxia region, this study still has some limitations. In our classification, some viruses are classified based on morphology or host specificity. This classification method is of certain significance for the initial understanding and differentiation of different viruses and cannot accurately reflect the systematic evolutionary relationship and essential differences between viruses. Although the sample collection covered sheep swab samples in the three provinces in a relatively comprehensive manner, the number of samples collected was limited, and only nasal swabs and anal swabs were collected. Therefore, subsequent studies can collect more comprehensive samples of sheep serum, gastrointestinal, and other sample materials so that the distribution of viruses in sheep can be studied more systematically.

## 5 Conclusion

In summary, this study systematically investigated the viruses carried by sheep in the Shaanxi-Gansu-Ningxia region and conducted a phylogenetic analysis of important viruses. The viral communities between the three provinces were not very different, but the number and proportion of reads of RNA and DNA viruses in each province were still different. Among the identified viruses are Astroviruses, Coronaviruses, Picornaviridae viruses, and Circoviruses, all of which can spread among humans and other mammals. Although we cannot explain the relationship between viruses of different species due to sample limitations, this study still comprehensively explored the basic situation of viruses carried by sheep in the Shaanxi-Gansu-Ningxia region, providing useful data for further research on the evolution and monitoring of such viruses.

## Data Availability

The datasets presented in this study can be found in online repositories. The names of the repository/repositories and accession number(s) can be found at: https://www.ncbi.nlm.nih.gov/, PRJNA1155242.

## Footnotes

^1^ https://www.stats.gov.cn/sj/ndsj/

^2^ http://tree.bio.ed.ac.uk/software/figtree
