# Supplementary material for: Metavirome analysis of domestic sheep in Shaanxi, Gansu, and Ningxia, China
Source: Front Vet Sci. 2024 Dec 3;11:1508617. doi: 10.3389/fvets.2024.1508617 (PMC11649628; doi:10.3389/fvets.2024.1508617)
Supplement: Supplementary file 1 [file Table_1.DOCX]

>Seq1 [organism=Goat astrovirus] [isolate=Caprine astrovirus-2023-Shannxi]

AGCACTGTCGATGAGGATGAGTGGCGCACTTTTGTGCTCACCACAAAGGGCTGTGAGCAC

ATCGCAGAAGAGGTCAACCACATACCTGACCACCAAGCCACAATCCTCACGGCGGCGTTG

ATGAATGATCGTATAAACCGGAAGAGGGAATTATCTGACCTGAAGTTGGAGAACCAGATT

CTGAGGGCGGAACTTGCACAACACCGCAATGAACAGGCAACCACAACAAGATTTGTCCTT

AGTTGGCCAACCATATTCGCCCTTGTGTTTCTGGGTTTCTCGCTGTTCTGGCATTCTGCC

CATGCCCTGACGACCACGTCGACGACAGATTCCACTGATCCAATGGGTATGCTTCGCCTT

AATATCTGGCTTGATGATTTTATCAAGAATGCGAAGAACACTATCCACACCCACCACACT

ACAGTAGTTGGCGTTATACAATCATCACCAACCTGGATTATGGTCAATCTTGTGATGCCG

TACATGTGGACTTTTGTCGTTATTTGTCTGGGTCTTATTTCTGTGTATAAATCAGAACAT

AAGGTTCTTTCCCTCTTGTTCTTGTGTGCTTCATCCCTCAGTGGAGGCGACTGGTTAATG

CTGTCCACAGCCTCAGCGCAGACAGTCCCGAGTGCCATTGCACAGGTTCTATGCGTCTTG

ATATCACACGTGGATCCATTGGGTTCTTTGTGCCTCTCAGCTGTTATTATGGGTGTGACC

TTTTTAACAAGCATGTGCATGTCGAATGTTAACTTTATACAACATTCAAGGGCATCAGCA

ATAAATACAGTCACATTAGTTATATCAGTCATGCTGAGGACACTCAAATTACCAGCTCTT

CCAATTGCGTGTGCACTGGCTCTTGTTAGGGCATACACAATCATGACATCCGCGAGTGGG

TCCACCATTGAAATCCGGTCTGAGGATGGCAAAGTGATCGCTAAGGAACCCGCCAAACCT

GGTGTTCTCTTCAGGTTCAAACAGGCTCTCCGTAGATTCGGCCAGGTCAGATCAAGCATA

GCTCCACTCGTTAGAGTCAACCCATCAGCAGTTGTTAGGATTGAGACCCCTGATGGAATT

GGGACTGGATTTGCCTGCGCGAACTACATCGTTACCGCAGGCCATGTGGTCGGCACACAC

AAGGCAGTCTCAGTTTGTGTCGGGAAGGCAAAGTACCAATCAACCCTTGTCCGCCACATT

GATGGTAAAGATGTCGCCCTCCTAAAAATGCCCCAGCAGCTTCAAGGCATGCCCCGCCTG

AAGATCGCCTCCAAGGTCGAGACGGAGTGGGTCTGCGTCTACTCACCTGATGAGGAAGGT

GCGATTGTTCAGTCTGTTGTGCCTGGTCACCAGATCGATGAGTGCATCAATTACGCTGTT

CCAACTCGTGATGGCATGTCTGGAGCACCTATTGTGAACCCCGATGGTAGAGTTATGGCG

GTCCACCTCACCAATACTGGCTACACCGGAGGAGCCGTTATCCTGACCCTCCAGGATGTA

ACTGATCCACCTAAGTCAAACCCCACTGAGGACAAACTCAAGGCTGAAATTGAGGAGCTT

AAGAAGCAGCTTGCGTCCTGCAACCAATCCAATACCTCTGAGCAG

>Seq2 [organism=Goat astrovirus] [isolate=Caprine astrovirus-2023-Gansu]

TTTTGACAGCTAGTACGGTCTGTGAGGAGGAGTGGCGGACTTTTGTGCTCACCACACAGG

GATGTGAGCACATAGCGGTCGAGGTGAACCATATACCCGATCATCAGCCTACTATCCTCA

CAGCGGCGCTTATGAACGACCGCGCCGACCGGAAGAGAGAACTATCTGACTTGCGCCTCG

AGAACCAACTGCTTAGAGCAGAGTTGGCCCAGCATCGTAATGAGGAAAAAACAACCACTA

AGTTTGTCTTGAGCTGGCCTACTATCTTTGCTTTGGTTTTTCTTGGCTTCTCCATGTTCT

GGCACTCTGCACATGGTCTCACTACCACATCAACAACTGATTCTATAGACCCTATGGGAA

TACTTAAGCTTAATGCATGGCTTGATGACTTTGTGAAGAACGCCATGAAGACGGTCCACA

CCCACCACACAACACTGGTTGGTGTTATACAATCTTCACCCACCTGGATTCTTGTGAACA

TGGTAATGCCATACATGTGGACCGTCGTTGTTTTGTGCCTCGGCTTGATATCAGTCTATA

AATCAGAGCACAAGGTTATATCCATTTTGTTCCTGTGCGCATCTACGTTGAGTGGTGGAG

ATTGGGTTATGCTAGCAACATCCTCATTCCAGACAATACCCAGTGCCATCGTCCAGATAA

TATGCGTCATGATATCACACATTGACGCCCTAGGTGCCATGTGCCTGTCTGTGGTTGTTA

TGTTCACCACTTTTCTAGCTAGCATGTGCCTGTCTAACATCAGTTTCATACAACACTCTC

GCGCGGCAGCCATAAATACTGCATCTCTTGTTGTTTCCATCGTTTTGAGGACACTTAGGT

TACCAGCAATGCCCCTTGCTATTGCCATGGCCCTCGTTAGAGCCTACACTATCCTCACAA

CAACAAATGGCTCAACAATTGAGGTGAGGTCTGAGGATGGCAAGGTTGTGTCGAAGGAAC

CCGCGAGGCCAGGCCTATTATTCAGGTTTAAGCAAGCATGCAGACGCTTCGCCCAGGTGA

GATCAAGCATGGCTCCACTTGTACGTGTCAATCCTGCCGCTGTCGTAAGGATTGAGACAC

CAGACGGCATTGGCACCGGCTTTGCATGTGCAAATTACATTGTGACAGCTGGTCATGTCC

TGGGTAATCATAAGGTCGCATCTGCATGTGTTGGTAAGGCTAAGTATCAGGCACCAATTG

CCAGACATGTTGAGGGCAAAGATGTTGTCCTCTTGAAAATGCCCCAGCAGCTCCAGAGCA

TGCCGCGGCTCAAGATAGCCTCCAAGATTGAAACTGACTGGGTGTGTGTATACTCACCAG

ATGATGAAGGTGCAATCGTCCAGTCAGTAGTTCCTGGCCATCAAGTTGAAGACTGTATAA

ACTATGCCGTGCCAACTCGTGATGGAATGTCTGGGGCCCCGATTGTCAACCCTGACGGGA

GAGTCATGGGTGTACACCTAACCAACACTGGGTACACTGGCGGAGCTGTTATCTTAGCTT

TACAGGATGTCACTGAT

>Seq3 [organism=Torovirus] [isolate=Goat torovirus 1-2022-Ningxia]

TGAATTCTATGCTTAATCCCAATGCTATGCCTTTTCAACCACCAACACAGGTTATGGCAATG

CCTGTTCAGTATCCTTTGGCATTCCAGCCTCGGTTTCGTAGGAGGCGAAACCCTGGTTTT

AGGCCTATGTTTCAGAGACGTGGTAATGGTGGGCAAAATTTGAGCCGCCAGAGTAGATCA

CGGTTTCAGAACCAGAGACGTGGTATTAATTCTCGACGTGGACAGCGTGGTGTTGGCCGT

CAGTCTAACAATCAGTCTGTTTCTATGCCATTTGAGCAACAATTGCTTATGATGGCAAAT

GAGACAGCTTCTTCTGCTGCATATCCACCTGAGTTGCAGAATTTTGCGCCCACCAAGCTT

GTGAAATTAGCTAAGCGGGCTGCTATGCAGATTGTTTCTGGCCATGCCACCGTTGAGGTC

TCGAGCGCCACTGATGAAACGTCTCATAAGATTGCAACTTTTACTTTAAAGGTTGCGCTA

AATTAA

>Seq4 [organism=Torovirus] [isolate=Goat torovirus 2-2022-Ningxia]

CAAATTTGCCCTTTGAACAGCAATTGCTGATGATGGCAAATGAAACCGCATATGCTGCAA

CTTATCCACCAGAGATGCAGACTGTTGCCCCCACTAAGTTAGTGAAAATTGCCAAAAGAG

CTGCAATGCAGATTGTCTCCGGTCATGCTACCGTTGAGATTTCCAATGGCACAGAAG

>Seq5 [organism=Torovirus] [isolate=Goat torovirus 3-2022-Ningxia]

ATCTTTAGAGAGAGAGCCAAGATGAATTCTATGCTTAATCCAAATGCTGTGCCATTTCAA

CCACCTACTCAGGTTGTCGCCATACCTATGCAGTATCCATCAGGCTTTCCTCCTGGGTTC

CGTAGACAACGCAACCTAGGTTTTCGGCCTATGTTTAATAGGCGGCGTAATAATAATGTT

AATCAAAACCGTGGCCGTCAAAACCGGCAACGTGTTCAAAATAATAATCGTGGCAATATT

GGAAATCGTCAAAATAACGGGCAACGCGGTAATAGACGTCAATATAATCAGGCGTCCCCA

AATTTGCCCTTTGAACAGCAATTGCTGATGATGGCAAATGAAACCGCATATGCTGCAACT

TATCCACCAGAGATGCAGACTGTTGCCCCCACTAAGT

>Seq6 [organism=Bovine viral diarrhea virus 2] [isolate=Bovine viral diarrhea virus 1-2022-Ningxia]

AGATTCCCCACAGCATTACTTAAAGTGAGAAGGGGGCTAGAAACGG

GATGGGCCTACACGCATCAAGGAGGGATCAGCTCGGTAGACCATGTCACAGCTGGAAAGG

ATTTACTGGTGTGTGACAGTATGGGTAGGACCAGGGTTGTCTGCCATAGTAACAATAAGA

TGACTGACGAGACTGAGTATGGCATCAAGACCGACTCTGGGTGTCCCGAAGGTGCAAGGT

GTTACGTGTTAAACCCAGAAGCCATAAACATTTCTGGCACAAAAGGAGCTATGGTACATC

TCCAGAAAACGGGGGGGGAGTTCACATGTGTCACTGCCTCAGGGACTCCGGCTTTCTTTG

ATCTGAAAAATCTAAAAGGTTGGTCTGGGCTACCAATTTTTGAAGCATCCAGTGGCAGGG

TGGTTGGTAGGGTGAAAGTCGGCAAAAATGAGGACTCCAAGCCCACCAAACTAATGAGTG

GGATCCAGACGGTGTCCAAGAACCAGACGGACCTAGCAGACATTGTAAAAAAATTGACCA

GCATGAACAGAGGAGAGTTCAAACAGGTAACACTGGCCACTGGGGCAGGAAAAACGACGG

AGCTGCCAAGGTCCGTCATAGAGGAGATAGGGAGACACAAAAGAGTCTTAGTCCTGATTC

CATTGAGAGCCGCTGCAGAGTCAGTGTACCAATATATGAGAGTGAAGTACCCAAGTATAT

CTTTCAATCTGAGAATAGGAGATATGAAAGAAGGTGACATGGCTACTGGTATCACTTATG

CCTCATATGGGTACTTTTGCCAGCTTCCTCAGCCCAAACTGAGAGCTGCCATGGTAGAGT

ACTCATATATATTCTTAGATGAGTATCACTGTGCTACACCTGAGCAATTAGCAATAATTG

GAAAGATACACAGATTTGCTGAAAATCTCAGAGTGGTAGCAATGACAGCAACCCCAGCTG

GCACGGTCACAACGACTGGTCAGAAACACCCTATAGAGGAGTTTATAGCCCCAGAGGTAA

TGAAGGGTGAAGATCTAGGAAGTGAATACTTGGATATTGCAGGGCTGAAGATACCAACTG

AAGAGATGAAAGGCAACATGCTCGTTTTCGTGCCAACTAGGAATATGGCAGTAGAAACGG

CTAAGAAATTGAAGGCAAAAGGGTACAACTCTGGATACTATTATAGTGGGGAAAACCCAG

AAAACTTGAGGGTGGTAACATCGCAATCCCCGTATGTGGTAGTAGCCACCAATGCCATAG

AGTCAGGTGTGACATTACCAGACTTAGACACAGTTGTAGACACTGGACTAAAGTGTGAGA

AGAGGGTGAGGATATCTTCAAAAATGCCCTTCATTGTAACAGGACTCAAGAGAATGGCAG

TCACCATAGGAGAGCAAGCCCAGCGCAGGGGTAGAGTAGGAAGAGTCAAGCCAGGTAGGT

ACTATAGAAGTCAGGAAACGGCTTCAGGGTCGAAAGATTACCATTACGACCTACTACAGG

CTCAGAGGTACGGAATAGAAGATGGAATTAATGTAACAAAGTCATTTAGGGAGATGAACT

ATGATTGGAGCCTTTATGAGGAGGACAGCCTGATGATAACTCAACTCGAGGTCCTCAACA

ACCTCCTTATATCAGAAGACCTGCCTGCCGCAGTGAAGAACATCATGGCTCGGACTGACC

ACCCAGAACCCATACAACTGGCCTATAACAGTTATGAAAATCAAATTCCAGTGCTATTCC

CAAAGATCAAAAATGGCGAGGTGACAGACACTTATGAGAATTACACATACCTCAATGCAA

GAAAACTAGGAGAGGACGTACCAGCGTATGTGTACGCTACAGAAGATGAGGATCTAGCAG

TGGACCTTCTAGGTATGGATTGGCCGGACCCAGGCAATCAGCAGGTGGTAGAGACAGGGA

GGGCGTTAAAACAAGTAACTGGCTTATCTACAGCAGAGAATGCCCTCTTGATAGCCCTGT

TCGGCTACGTCGGGTACCAGACACTTTCAAAAAGGCATATACCAATGGTCACCGACATCT

ATACGCTTGAAGACCATAGGCTGGAGGACACAACCCACCTCCAGTTTGCCCCAAATGCTA

TAAGGACTGACGGCAAGGACGCAGAGTTGAAGGAATTAGCCGTCGGAGACCTTGATAAAT

ATGTGGACGCACTGGTGGACTACTCCAAACAAGGGATGAAGTTTATCAAAGTCCAAGCTG

AAAAGGTCAAGGACTCCCATTCTACAAAGGAAGGCCTGCAAACAATCAAGGAGTATGTGG

ATAAGTTCATACAATCACTAATAGAGAACAAGGAGGAGATCATCAGGTATGGACTATGGG

GAGCTCACACAGCACTCTACAAAAGCTTGGCAGCGAGATTGGGGCATGAAACAGCTTTTG

CAACTTTAGTGGTAAAATGGTTGGCTTTTGGGGGCGAAACGGTATCAGCCCACATTAAGC

AAGCAGCAGTTGATCTAGTAGTGTATTATATCATGAACAAACCATCTTTTCCTGGAGACA

CAGAGACCCAACAAGAGGGGAGGAGGTTTGTAGCTAGTCTTTTCATATCTGCGCTAGCGA

CATACACATACAAAACCTGGAATTACAACAATCTGTCACGGGTCGTTGAGCCTGCCTTAG

CTTACCTCCCATATGCTACAAGTGCCTTGAAGTTGTTCACACCTACAAGACTAGAGAGTG

TGGTCATACTCAGTTCTACAATCTACAAGACGTACCTCTCCATAAGGAAGGGCAAGAGCG

ACGGCCTACTGGGTACAGGTATAAGTGCAGCCATGGAGATCTTAAACCAAAACCCAATCT

CAGTTGGTATATCTGTGATGCTGGGGGTGGGTGCTATTGCCGCCCATAATGCAATAGAAT

CCAGTGAACAGAAAAGAACTTTGTTGATGAAAGTCTTTGTAAAAAACTTCTTGGACCAGG

CAGCAACAGATGAGCTAGTAAAAGAGAACCCCGAAAAAATAATCATGGCTCTGTTTGAAG

CAGTCCAGACCATAGGCAACCCCCTAAGACTCATCTACCATCTGTACGGGGTGTACTATA

AGGGGTGGGAAGCAAAAGAACTCGCAGAAAAAACTGCCGGCCGTAACTTATTTACACTGA

TCATGTTTGAAGCCTTTGAACTTTTGGGTATGGACTCAGAAGGGAAGATAAGAAACTTGT

CGGGTAACTACATACTGGACTTGATCTTCAATTTGCATAATAAATTAAACAAGGGACTCA

AAAAGTTGGTCCTTGGGTGGGCCCCAGCGCCTTTTAGCTGCGATTGGACACCTAGTGTTG

AGAGGTTAAGCTTGCCTCACAACAACTACTTAAGGTTGGAAACCAAGTGTCCTTTCGGCT

ATGAGATGACGGCAATAAAAATCGTTGCTGGCTAGTTGACAATAGTTGAAGAGAAGGGGT

CCTTCTTCTGCTGAGACAGTTTAGGGAGAGGACCTCCAAATTTCAAAGTAACAACATTCT

TTGATGACTCTTTGATAGAAGTCATGCCAGTATCTAGGCCAGAAGGCCACGTGGATCTCT

ACTACAAGGGAGTAACAGCAAGGTTAGACTATAGTAACGGGAAAGTGCTGTTAGCTACCA

ACAAGTGGGAGGTGGACCACGCCTTTCTGACTAGACTAGTAAAGAAGCACACAGGGATAG

GTTTTAAAGGTGCATATTTGGGTGACCGACCAGACCATCAAGATCTTGTCAGTAGAGATT

GTGCAACCATAACGAAGAACTCAGTACAGTTTCTGAAAATGAAGAAGGGTTGCGCTTTTA

CATACGACCTAACAATCTCTAACCTTGTCAGGCTTATTGAACTAGTCCACAAGAATGATT

TACAAGAGAGAGAAATCCCGACTGTGACAGTAACTACCTGGCTTGCTTATTCTTTTGTCA

ACGAAGACCTGGGGACTATCAAGCCTGTGTTGGGGGAGAAAGTCATCCCAGAGCCCCCCA

TGGAGTTGAGTCTTCAACCCGCTGTGGAGCTGGTTACCACTGAAACAGCGATAACCATAA

CAGGGGAGGCTGAAGTGATGACGACAGGGATCACACCGGTGGTAGAAATGAAAGAAGAAC

CTCAGCTGGGCCATCAGTCAACTACCCTAAAGGTAGGATTGAAGGAAGGGGAATTCCCAG

GGCCAGGAGTCAACCCTAACCATTTAGTAGAGGTGATAGATGAGAAAGATGTCAGGCCTT

TTGTCCTAGTTATCGGGAACAAAGGTTCTACCTCAAATAGAGCAAAAACAGCCAAGAATA

TACGTTTGTACAAAGGAAACAATCCAAGAGAGATCAGGGATCTGATGAGCCAAGGGAGAA

TATTAACGGTTGCTCTAAAAGAGTTGGACCCGGAACTAAGAGAGTTGGTAGATTATAAGG

GGACCTTTCTCAATAGGGAAGCTTTAGAAGCCTTAAGCTTAGGTAAACCAATTAAGAGGA

AAACCACAACAGCAATGATCAGGAGGTTGATAGAGCCAGAGGCTGAGGAGGAACTACCAG

ATTGGTTCCAAGCGGAAGAACCCCTATTTTTGGAAGCAAGAATCCAGGCTGACATTTATC

ACCTGGTGGGCAGTGTTGACAGTATAAAAAGCAAAGCAAAGGAATTGGGGGCCACAGATA

ACACAAAGATTGTGAAGGAGGTCGGGGCTAGGACCTATACTATGAAATTGAGCAGTTGGA

GTACACAAGTTATCAATAAACAAATGAGTCTAGCCCCCCTTTTTGAAGAGCTGTTATTAA

AGTGTCCTCCATGTAGCAAAATTTCAAAGGGACATATGGTGTCAGCATACCAACTGGCCC

AAGGAAACTGGGAACCCCTCGGGTGCGGGGTTTATATGGGGACTGTACCAGCTAGGCGTC

TCAAGATCCACCCTTACGAAGCCTACCTCAAACTCAAAGAGCTGGTGGAAGGTGAACTTT

CGAGGGTTACCGCAAGAGAATCCATCATAAGAGAACATAACACCTGGATTTTGCGGAAAG

TGAGACACGAAGGGAACCTAAGAACTAAATCAATGATTAACCCTGGGAAAGTATCAGAAC

AGTTGTGCAGAGAAGGACACAAAAGAAACATATACAATAAGATTATAGGCTCAACAATGG

CCTCTACTGGTATCAGGCTGGAGAAACTGCCAATAGTCCGAGCCCAAACTGATACAACCA

GTTTCCATCAAGCTATAAGAGAGAAAATTGATAAGCCAGAAAACAAGCAAACCCCTGAAT

TGCATGAAGAACTAAAGAAGGTTTTCGACTGCTTAAAGATCCCGGAGTTGAAAGAATCGT

ATGATGAAGTTTCATGGGAACAATTAGAAGCTGGGATAAACCGCAAGGGAGCAGCCGGTT

TTCTAGAGAGTAAGAACATAGGGGAGGTGCTGGACACAGAAAAACACATAGTGGAACAAC

TAATCAGGGATTTGAGGCAGGGGAAGAAGATCAGGTATTATGAAACAGCCATCCCCAAGA

ATGAGAAGAGAGACGTCAGTGACGACTGGGAAGCCGGAGAATTTGTTGATGAAAAGAAAC

CAAGAGTAATCCAGTACCCGGATGCCAAGGTGAGACTGGCCATCACAAAAGTAATGTACA

AGTGGGTAAAGCAACAACCAGTGGTGATACCAGGCTATGAGGGTAAAACACCGCTATTTG

ATATATTCAACAAGGTGAAGAAGGAATGGGATTCATTCCAGGACCCCGTAGCGGTGAGCT

TTGACACCAAAGCCTGGGATACACAAGTTACTAGTAGAGACCTAATGTTGATAAGGGACA

TCCAGAAATATTATTTCAAGAAAAACATACACAAATTCTTAGATACAATAACAGAACACA

TGGTGGAGGTACCCGTCATTACAGCAGACGGTGAAGTTTACATAAGGAATGGTCAGAGGG

GTAGTGGCCAACCCGACACAAGTGCTGGTAACAGTATGTTGAATGTCCTAACCATGATAT

ATGCCTTCTGTAAAAGCACAGGTATACCTTACAGGGGATTCAGCAGAGTGGCAAGAATCC

ATGTGTGTGGTGATGATGGCTTCCTGATAACAGAAAGAGGACTGGGGTTGAAATTCTCTG

AGAAGGGTATGCAGATATTACATGAGGCCGGGAAGCCCCAGAAAATAACTGAAGGGGACA

AAATGAAAGTGGCATACAGATTCGAGGACATTGAGTTCTGTTCCCATACTCCTGTACCAG

TCAGATGGGCAGATAACACCAGTAGTTACATGGCAGGAAGGAGTACAGCCACTATACTAG

CTAAGATGGCAACCAGGCTGGATTCCAGCGGAGAGAGGGGTAGCACAGCTTATGAGAAGG

CCGTAGCCTTTAGCTTCCTTTTGATGTACTCATGGAATCCGGTGGTTAGAAGGATCTGCT

TAATGGTGCTGTCACAGTATCCGGAAGTATCCCCATCCAAACATACAATATACTACTACC

AAGGGGATCCCATAGCTGCATACAGAGAAGTGATAGGGAGACAGCTGTGTGAACTGAAAA

GAACAGGATTTGAGAAGCTAGCTAGTCTGAACCTGAGTATGACCACTCTAGGCATCTGGA

CAAAACATACTAGTAAAAGACTAATCCAAGACTGTGTAGAAATAGGTAAGAGAGAAGGTA

ATTGGTTAGTTAATGCTGACAGACTGATTTCAGGAAAGACTGGGAAGTTTTACATTCCAA

ACACTGGTGTCACTCTGTTAGGAAAACATTATGAGGAAATTAACTTAAAGCAAAAGGCGG

CACAACCGCCGACGGAAGGGGTTGACAGATATAAGTTAGGCCCCATAGTTAATATTATTT

TGAGAAGATTGAGGGTGATGCTAATGACAGCGGCCAGCGGAAACTGGTAA

>Seq7 [organism=Bovine viral diarrhea virus 2] [isolate=Bovine viral diarrhea virus 2-2022-Ningxia]

ATGGAGTTGTTTTCAAATGAACTTTTATACAAAACATATAAACAAAA

ACCAGAAGGTGTTGTGGAACCTGTTTACGACATCAACGGGTGTCCATTGTTTGGAGAGAG

CAGTGACGTACACCCGCAATCCACTCTAAAACTACCACACCAACGAGGCAGCGGTAACAT

CCTGACCAATGCTAGGTCCCTGCCGCGTAAAGGTGACTGCCGGAAAGGCAACGCAAATGG

AGCGGTGAGTGGTATCTACATTAAACCAGGACCAATCTACTATCAGGATTATGTGGGGCC

CGTCTATCATAGAGCCCCACTGGAACTATGTAGAGAGGCAAGCATGTGTGAAACTACTAG

GAGAGTCGGCAGAGTGACCGGTAGTAACGGGAAATTATACCACATCTATATTTGCATAGA

CGGGTGTATCCTCCTGAAGAGGGCAACTAGGAACCAACCAGAAGTCCTCAAATGGGTATA

CAACAGATTAAATTGCCCATTATGGGTCACCAGCTGCTCCGGTGAAGAGAGTAAGGGTGC

TACAAGCAAGAAGCAACCTAAGCCAGATAGGATAGAAAAAGGCAAGATGAAAATAGCCCC

TAAAGAGACAGAAAAAGATTGCAAAACCAGACCCCCTGACGCAACCATAGTAGTAGAAGG

GGTTAAGTACCAGGTGAAGAAGAAGGGAAAAGTAAGGAATAAAAATACTCAAGATGGGTT

GTATCACAATAAGAATAAGCCCCCTGAATCAAGAAAGAAATTGGAGAAGGCACTGCTAGC

ATGGGCCATCTTAGCAGCAGTCCTGCTTCAGCTGGTAACGGGAGAGAACATTACCCAGTG

GAACTTGATGGACAACGGCACCGAAGGTATACACCAAGCTATGTTCCTGAGAGGGGTGAA

CAGGAGTCTACATGGAATTTGGCCCGAGAAGATTTGCACCGGAGTACCAACTCACTTAGC

AACAGACTACGAGCTCAAGGAGATAGTGGGAATGATGGACGCGAGTGAGAAGACCAACTA

CACATGTTGCAGGTTGCAAAGGCATGAGTGGAACAAACATGGCTGGTGCAATTGGTTTCA

TATAGAACCGTGGATATGGCTGATGAACAAAACCCAAAGCAACTTAACTGAAGGACAGCC

ATTTAGGGAGTGTGCTGTAACTTGTAGGTATGACAGGGAAACAGAATTGAACATCGTAAC

ACAGGCTAGGGACAGGCCTACAACTCTGACAGGTTGCAAGAAAGGCAAGAATTTTTCCTT

TGCGGGGGTTGTACTGGATGGGCCCTGCAACTTTAAAGTATCAGTTGAAGACGTGCTGTT

CAAGGAACACGATTGTGGCAACATGCTACAGGAGACCACGATACAGCTACTCGATGGGGC

AACCAACACCATTGAGGGAGCAAGGGCAGGGACGGCCAAGTTGACAACCTGGTTAGGGAA

GCAATTAGGGATCCTTGGTAAGAAGTTGGAGAATAAAAGCAAAGCATGGTTTGGTGCATA

CGCAGCAAGCCCATACTGTGAAGTGGAGAGGAAGATCGGCTACATCTGGTATACAAAAAA

CTGCACTCCAGCTTGCCTTCCAAGAAACACTAAGATAATAGGCCCCGGGAAGTTCGATAC

CAACGCTGAAGATGGCAAAATACTCCATGAGATGGGAGGGCACCTCTCAGAATTTACCCT

ATTGTCCTTGGTGGTTCTGTCTGACTTTGCCCCAGAAACCGCGAGTGTTATCTACTTGGT

GCTACATTTCGCGATCCCGCAAAGTCACGTCAACATAGACACATGCGACAAGAGCCAGCT

AAATTTAACGGTCGCAACTACAGTAGCAGAAGTCATACCAGGGACAGTGTGGAACCTAGG

AAAGTATGTCTGCATAAGACCAGACTGGTGGCCATATGAAACGACCACAGTCTTTGTTTT

AGAGGAAGCAGGACAAGTAATTAAATTGGGGCTAAGGGCCATCAGAGACTTAACCAGGAT

TTGGAACGCTGCCACCACAACAGCCTTCCTAGTCTTCCTTGTGAAAGTACTGAGGGGACA

ACTAATCCAAGGGCTATTATGGCTGATGTTAATAACAGGGGCACAGGGCTTCCCTGAATG

CAAAGGGGGCTTCCGATATGCCATATCAAAAGACAGAAAAATAGGACCACTGGGGCCAGA

GAGTTTAACTACAACATGGCACCTTCCTACCAAAAAAATAGTGGACTCCATGGTACAGGT

GTGGTGTGATGGAAAAAACTTGAAAATATTAAAAACGTGCACAAAGGAAGAGAGGTACTT

AGTGGCCGTGCACAAAAGAGCCCTACCAACCAGTGCTGAGTTCATGCAGATCAGTAGTGG

GACAAAAGGCCCAGAAGTGATAGATATGCATGATGACTTTGAATTTGGACTCTGTCCTTG

TGATTCAAAACCGGTAGTAAGGGGGAAGTTCAATGCCAGTTTATTGAATGGACCAGCTTT

CCAGATGGTATGCCCACTGGGGTGGACTGGTACAATAGAATGCATCCTGGCAAACCAAGA

CACCTTGGACACAACTGTCGTTAGGACATATAGAAGAACTACTCCATTTCAGCGGAGAAA

ATGGTGTACCTATGAAAAGATAATAGGGGAGGATATCCATGAATGCATTCTGGGAGGAAA

CTGGACATGCATAACTGGTGACCATAGCAAGTTGAAAGATGGGCCCATCAAGAAGTGTAA

GTGGTGTGGCTACGACTTCTTCGATTCAGAAGGACTGCCGCACTACCCAATAGGTAAGTG

CATGCTCAGCAACGAGAGTGGGTACAGGTATGTAGATGACACCTCTTGTGATAGGGGTGG

TGTAGCCATAGTTCCAACAGGTACCTTAAAGTGTAGAATAGGCAACACCACGGTGCAGGT

TATCGCTACTAACACTGACCTGGGACCCATGCCCTGCAGCCCAGATGAGGTGATAGCAAG

TGAAGGACCAGTGGAAAAGACGGCATGCACGTTTAATTATTCAAAGACACTACCTAATAA

GTATTATGAACCGAGGGACCGGTACTTCCAACAATACATGTTAAAAGGGAAGTGGCAATA

TTGGTTTGACCTGGATACTGTAGACCACCACAAAGACTACTTTTCAGAGTTCATAGTCAT

AGCAGTGGTAGCCTTACTAGGTGGTAAGTATGTACTGTGGCTCTTAGTAACATATATGAT

ACTGTCTGAGCAGATGGCTATGGGTGCTAGAGTAAGTACCGAAGAGATAGTCATGATAGG

CAACTTGTTGACACACAGTGACATCGAGGTTGTGGTCTATTTTCTTCTTTTGTACTTAAT

AATCAAAGAGGAACTGGTGAGGAAATGGATTATACTAGTGTACCACATCCTTGTAGTAAA

TCCTATGAAAACAATAGGAGTTATCCTACTAATGCTAGGGGGGGTGGTGAAGGCCAGCAA

AATCAATACTGATGACCAGAGTGCTATGGACCCATGTTTTCTTCTCGTAACAGGCTTAGT

GGCCGTTTTGATGATCGCTAGAAGAGAACCTGCCACCTTTCCGCTGGTTGCAGCATTAT

>Seq8 [organism=Bovine viral diarrhea virus 2] [isolate=Bovine viral diarrhea virus 3-2022-Ningxia]

GGGGGGTGGTGAAGGCCAGCAAAATCAATACTGATGACCAGAGTGCTATGGACCCATGTT

TTCTTCTCGTAACAGGCTTAGTGGCCGTTTTGATGATCGCTAGAAGAGAACCTGCCACCT

TTCCGCTGGTTGCAGCATTATTGGCAATAAGAACATCAGGATTCCTACTACCCGCTAGCA

TTGATATAACTGTAGCAGTAGTGCTAATCGTACTTCTGCTAGCTAGCTACTTAACAGACT

ATTTCAGATATAAAAAGTGGCTTCAATTTTCATTTAGTCTGATAGCTGGTATTTTTGTCA

TAAGGAGTTTGAAACATATCAACCAGATGGAGGTACCAGAAATATCTATGCCAAGTTGGA

GACCTCTAGCCCTTGTTATTTTCTATATAATATCTACAGCTATAACCACTAGTTGGGACA

TTGACTTAGCAGGCTTCCTGCTGCAATGGGCGCCAGCAGTGATCATGATGGCCACCATGT

GGGCAGACTTTTTGACTCTAATCATAGTCCTACCCAGTTACGAGCTGTCTAAGCTTTACT

TCCTGAAGAATGTCAGGACTGATGTAGAAAAGAACTGGCTCGGCAAGGTGAAATACAGAC

AGATCAGCTCAGTTTATGATATCTGTGACAGTGAAGAAGCAGTATACCTATTTCCATCAA

GGCATAAGAGCGGGAGCAGACCAGATTTTGTATTACCTTTTTTAAAAGCCGTGTTAATAA

GCTGCATCAGTAGCCAATGGCAGGTGGTCTACATTTCCTACCTAATACTGGAAATTACAT

ACTATATGCACAGGAAAATCATAGATGAGGTGTCAGGAGGTGCAAATTTCTTGTCAAGAC

TTATAGCAGCCACCATAGAATTAAACTGGGCCATTGATGATGAGGAATGTAAAGGACTGA

AGAAATTATATCTCTTATCAGGGAGAGTAAGGAATTTGATAGTTAAACACAAGG

>Seq9 [organism=Bovine coronavirus] [isolate=Goat coronavirus-2023-Shannxi]

ATGTTTTTGATACTTTTAATTTCCTTACCAACGGCTTTTGCTGTTATAGGAGATTTAAAG

TGCACTACAGTTTCCATTAATGATGTTGACACTGGTGTTCCTTCTATTAGCACTGATACT

GTCGATGTTACTAATGGTTTAGGTACTTATTATGTTTTAGATCGTGTGTATTTAAATACT

ACGTTGTTGCTTAATGGTTACTACCCCACTTCAGGTTCTACATATCGTAATATGGCACTG

AAGGGAACTTTACTATTGAGCACACTATGGTTTAAACCACCTTTTCTTTCTGATTTTACT

AATGGTATTTTTGCTAAGGTCAAAAATACCAAGGTTATTAAAGATGGTGTAATGTATAGT

GAGTTTCCTGCTATAACTATAGGTAGTACTTTTGTAAATACATCCTATAGTGTGGTAGTA

CAACCACATACTACCAATTTGGATAATAAATTACAAGGTCTCTTAGAGATCTCTGTTTGC

CAGTATACTATGTGCGAGTACCCACATACGATTTGTCATCCTAACCTGGGTAATCAACGC

GTAGAACTATGGCATTGGGATACAGGTGTTGTTTCCTGTTTATATAAGCGTAATTTCACA

TATGATGTGAATGCTGATTATTTGTATTTCCATTTTTATCAAGAAGGTGGTACTTTTTAT

GCATATTTTACAGACACTGGTGTTGTTACTAAGTTTCTGTTTAATGTTTATTTAGGCACG

GTGCTTTCACATTATTATGTCATGCCTTTGACTTGTAATAGTGCTATGACTTTAGAATAT

TGGGTTACACCTCTCACTTCTAAACAATATTTACTAGCTTTCAATCAAGATGGTGTTATT

TCTAATGCTGTTGATTGTAAGAGTGACTTTATGAGTGAGATTAAGTGTAAAACACTATCT

ATAGCACCATCTACTGGTGTTTATGAATTAAACGGTTACACTGTTCAGCCAATTGCAGAT

GTTTACCGACGTATACCTAATCTTCCCGATTGCAATATTGAGGCTTGGCTTAATGATAAG

TCGGTGCCGTCTCCACTAAATTGGGAACGTAAAACCTTTTCAAATTGTAATTTTAATATG

AGCAGCCTGATGTCTTTTATTCAGGCAGACTCATTTACTTGTAATAATATTGATGCTGCT

AAGATATATGGTATGTGTTTTTCCAGCATAACTATAGATAAGTTTGCTATACCCAATGGT

AGGAAGGTTGACCTACAATTGGGTAATTTGGGCTATTTGCAGTCTTTTAACTATAGAATT

GATACTACTGCTACAAGTTGTCAGTTGTATTATAATCTACCCGCTGCTAATGTTTCTGTT

AGCAGGTTTAATCCTTCTACTTGGAATAGGAGATTTGGTTTTACAGAACAATCTGTTTTT

AAGCCTCAACCTGCAGGTGTTTTTACTGATCATGATGTTGTCTATGCACAACATTGTTTT

AAAGCCCCCACCACTTTTTGTCCATGTAAATTGGATGGTTCACTTTGTGTTGGTAGTGGT

CCTGGTATTGATGCTGGTTATAAACATAATGGTATAGGTACTTGCCCTGCAGGTACTAAT

TATTTAACATGTTACAATTCTATTCAATGTAACTGTTTATGTGCACCTGATCCCATTACA

TCCACAACCACAGGTCCCTATAAATGCCCCCAAACTAAATATTTAGTTGGTGTAGGAGAG

CATTGTTCGGGTCTTGCTATTAATAGTGATCATTGTGGAGGCAATCCTTGTACTTGCCAA

CCACAAGGATTTTTGGGTTGGTCTGTGGATTCCTGTTTACAAGGAGACAGGTGTAATATT

TTTGCTAATTTAATTTTGCATGGTGTTAATAGTGGTACTACCTGTTCCACTGATTTACAA

AAAGCTAACACAGACATAATTCTTGGTGTTTGTGTTAATTATGATCTTTATGGTATTACT

GGCCAAGGTATTTTTGTTGAGGTTAATGCGACTTATTATAATAGTTGGCAGAACCTTTTA

TATGATTCTAATGGTAATCTCTATGGTTTTAGAGACTACTTAACAAATAGAACTTTTATG

ATTCGTAGTTGCTACAGCGGTCGTGTTTCAGCGGCCTTTCATGCTAACTCTTCCGAACCA

GCATTGCTATTTCGGAATATTAAATGCAATTACGTTTTTAATAATACTCTTTCACGAGAG

CTGCAACCTATTAATTATTTTGATAGCTATCTTGGTTGTGTTGTCAATGCTGATAATAGT

ACTTCTAGTGTTGTTCAAACATGTGATCTCACAGTAGGTAGTGGTTACTGTGTGGATTAC

TCTACAAAAAGACGAAGTCGTAGAGCGATTACCACTGGTTATCGGTTTACTAATTTTGAG

CCTTTTACTGTTAATGCAGTAAATGACAGTTTAGAACCTGTAGGTGGTTTGTATGAAATT

CAAATACCTTCAGAGTTTACTATAGGTAATATGGAGGAGTTTATTCAAACAAGCTCTCCT

AAAGTTACTATTGATTGTGCGGCTTTTGTCTGTGGTGATTATGCAGCATGTAAATTACAG

TTGGTTGAGTATGGTAGTTTTTGTGATAATATAAACGCCATACTCACAGAAGTAAATGAG

TTACTTGACACTACACAGTTGCAAGTTGCTAATAGTTTAATGAATGGCGTCACTCTTAGC

ACTAAGCTTAAAGATGGCGTTAATTTCAATGTAGATGACATCAACTTCTCCCCTGTATTA

GGTTGTTTAGGAAGTGATTGTAATAAAGCTTCCACTAGATCTGCTATAGAGGATTTACTT

TTTGATAAAGTTAAATTGTCTGATGTCGGTTTTGTTGAGGCTTATAATAATTGCACTGGA

GGTTCCGAAATTAGGGATCTCATTTGTGTTCAAAGTTATAATGGCATCAAAGTGCTACCT

CCATTATTATCTGAAAATCAGATTAGTGGTTACACTTTGGCTGCCACCTCCGCTAGTTTG

TTTCCACCATGGACAGCAGCGGCGGGTGTTCCATTTTATTTAAATGTTCAGTATCGTATT

AATGGACTTGGCGTTACTATGGATGTTTTAAGCCAAAACCAAAAGCTTATTGCTAATGCA

TTTAACAATGCTTTGACAGCTATTCAGAATGGGTTTGATGCAACCAATTCTGCTTTAGTT

AAAATTCAGTCTGTTGTTAATGCAAATGCTGAAGCTCTTAATAAGTTATTGCAACAACTA

TCTAATAGATTTGGAGCCATAAGTGCTTCTTTACAAGAAATTCTATCTAGGCTCGATGCT

CTTGAAGCACAAGCCCAGATAGATAGACTTATTAATGGTCGTCTTACCGCCCTTAATGCT

TATGTTTCTCAACAGCTTAGTGATTCTACACTAGTAAAATTTAGTGCAGCACAAGCTATG

GAGAAGGTTAATGAGTGTGTTAAAAGCCAATCATCTAGGATAAATTTTTGCGGTAATGGT

AATCATATTATATCATTAGTGCAGAATGCTCCTTATGGTTTGTATTTTATCCACTTTAAC

TATGTCCCCACCAAGTATGTCACAGCAAAGGTTAGTCCCGGTCTGTGCATTGCAGGTGAT

AGAGGTATAGCTCCTAAGAGTGGTTATTTTATTAATGTAAATAATACTTGGATGTTCACT

GGTAGTGGTTATTACTACCCTGAACCCATAACTGGAAATAATGTTGTTGTTATGAGCACC

TGTGCTGTTAATTATACTAAAGCACCGGATGTAATGCTGAACACTTCAGTACCCAACCTC

CCTGATTTTAAGGAAGAGTTGGATCAATGGTTTAAAAACCAAACATCAGTGGCACCAGAT

CTGTCACTTGATTATATAAATGTTACATTCTTGGACCTACAAGATGAAATGAATAGGTTA

CAGGAGGCAATAAAAGTCTTAAATCAGAGCTACATTAATCTCAAGGACATTGGTACATAT

GAATATTATGTAAAATGGCCTTGGTATGTATGGCTCTTAATAGGCCTTGCTTCTGTTGCT

ATGCTTGTTTTACTATTCTTCATATGCTGTTGTACAGGATGTGGGACTAGTTGTTTTAAG

AAATGTGGTGGTTGTTGTGATGATTATACTGGACACCAGGAGTTAGTAATTAAAACCTCA

CATGACGAC

>Seq10 [organism=Bovine coronavirus] [isolate=Goat coronavirus-2023-Gansu]

ATGTTTTTGATACTTTTAATTTCCTTACCAACGGCTTTTGCTGTTATAGGAGATTTAAAG

TGCACTACAGTTTCCATTAATGATGTTGACACTGGTGTTCCTTCTATTAGCACTGATACT

GTCGATGTTACTAATGGTTTAGGTACTTATTATGTTTTAGATCGTGTGTATTTAAATACT

ACGTTGTTGCTTAATGGTTACTACCCCACTTCAGGTTCTACATATCGTAATATGGCACTG

AAGGGAACTTTACTATTGAGCACACTATGGTTTAAACCACCTTTTCTTTCTGATTTTACT

AATGGTATTTTTGCTAAGGTCAAAAATACCAAGGTTATTAAAGATGGTGTAATGTATAGT

GAGTTTCCTGCTATAACTATAGGTAGTACTTTTGTAAATACATCCTATAGTGTGGTAGTA

CAACCACATACTACCAATTTGGATAATAAATTACAAGGTCTCTTAGAGATCTCTGTTTGC

CAGTATACTATGTGCGAGTACCCACATACGATTTGTCATCCTAACCTGGGTAATCAACGC

GTAGAACTATGGCATTGGGATACAGGTGTTGTTTCCTGTTTATATAAGCGTAATTTCACA

TATGATGTGAATGCTGATTATTTGTATTTCCATTTTTATCAAGAAGGTGGTACTTTTTAT

GCATATTTTACAGACACTGGTGTTGTTACTAAGTTTCTGTTTAATGTTTATTTAGGCACG

GTGCTTTCACATTATTATGTCATGCCTTTGACTTGTAATAGTGCTATGACTTTAGAATAT

TGGGTTACACCTCTCACTTCTAAACAATATTTACTAGCTTTCAATCAAGATGGTGTTATT

TCTAATGCTGTTGATTGTAAGAGTGACTTTATGAGTGAGATTAAGTGTAAAACACTATCT

ATAGCACCATCTACTGGTGTTTATGAATTAAACGGTTACACTGTTCAGCCAATTGCAGAT

GTTTACCGACGTATACCTAATCTTCCCGATTGCAATATTGAGGCTTGGCTTAATGATAAG

TCGGTGCCGTCTCCACTAAATTGGGAACGTAAAACCTTTTCAAATTGTAATTTTAATATG

AGCAGCCTGATGTCTTTTATTCAGGCAGACTCATTTACTTGTAATAATATTGATGCTGCT

AAGATATATGGTATGTGTTTTTCCAGCATAACTATAGATAAGTTTGCTATACCCAATGGT

AGGAAGGTTGACCTACAATTGGGTAATTTGGGCTATTTGCAGTCTTTTAACTATAGAATT

GATACTACTGCTACAAGTTGTCAGTTGTATTATAATCTACCCGCTGCTAATGTTTCTGTT

AGCAGGTTTAATCCTTCTACTTGGAATAGGAGATTTGGTTTTACAGAACAATCTGTTTTT

AAGCCTCAACCTGCAGGTGTTTTTACTGATCATGATGTTGTCTATGCACAACATTGTTTT

AAAGCCCCCACCACTTTTTGTCCATGTAAATTGGATGGTTCACTTTGTGTTGGTAGTGGT

CCTGGTATTGATGCTGGTTATAAACATAATGGTATAGGTACTTGCCCTGCAGGTACTAAT

TATTTAACATGTTACAATTCTATTCAATGTAACTGTTTATGTGCACCTGATCCCATTACA

TCCACAACCACAGGTCCCTATAAATGCCCCCAAACTAAATATTTAGTTGGTGTAGGAGAG

CATTGTTCGGGTCTTGCTATTAATAGTGATCATTGTGGAGGCAATCCTTGTACTTGCCAA

CCACAAGGATTTTTGGGTTGGTCTGTGGATTCCTGTTTACAAGGAGACAGGTGTAATATT

TTTGCTAATTTAATTTTGCATGGTGTTAATAGTGGTACTACCTGTTCTACTGATTTACAA

AAAGCTAACACAGACATAATTCTTGGTGTTTGTGTTAATTATGATCTTTATGGTATTACT

GGCCAAGGTATTTTTGTTGAGGTTAATGCGACTTATTATAATAGTTGGCAGAACCTTTTA

TATGATTCTAATGGTAATCTCTATGGTTTTAGAGACTACTTAACAAATAGAACTTTTATG

ATTCGTAGTTGCTACAGCGGTCGTGTTTCAGCGGCCTTTCATGCTAACTCTTCCGAACCA

GCATTGCTATTTCGGAATATTAAATGCAATTACGTTTTTAATAATACTCTTTCACGAGAG

CTGCAACCTATTAATTATTTTGATAGCTATCTTGGTTGTGTTGTCAATGCTGATAATAGT

ACTTCTAGTGTTGTTCAAACATGTGATCTCACAGTAGGTAGTGGTTACTGTGTGGATTAC

TCTACAAAAAGACGAAGTCGTAGAGCGATTACCACTGGTTATCGGTTTACTAATTTTGAG

CCTTTTACTGTTAATGCAGTAAATGACAGTTTAGAACCTGTAGGTGGTTTGTATGAAATT

CAAATACCTTCAGAGTTTACTATAGGTAATATGGAGGAGTTTATTCAAACAAGCTCTCCT

AAAGTTACTATTGATTGTGCGGCTTTTGTCTGTGGTGATTATGCAGCATGTAAATTACAG

TTGGTTGAGTATGGTAGTTTTTGTGATAATATAAACGCCATACTCACAGAAGTAAATGAG

TTACTTGACACTACACAGTTGCAAGTTGCTAATAGTTTAATGAATGGCGTCACTCTTAGC

ACTAAGCTTAAAGATGGCGTTAATTTCAATGTAGATGACATCAACTTCTCCCCTGTATTA

GGTTGTTTAGGAAGTGATTGTAATAAAGCTTCCACTAGATCTGCTATAGAGGATTTACTT

TTTGATAAAGTTAAATTGTCTGATGTCGGTTTTGTTGAGGCTTATAATAATTGCACTGGA

GGTTCCGAAATTAGGGATCTCATTTGTGTTCAAAGTTATAATGGCATCAAAGTGCTACCT

CCATTATTATCTGAAAATCAGATTAGTGGTTACACTTTGGCTGCCACCTCCGCTAGTTTG

TTTCCACCATGGACAGCAGCGGCGGGTGTTCCATTTTATTTAAATGTTCAGTATCGTATT

AATGGACTTGGCGTTACTATGGATGTTTTAAGCCAAAACCAAAAGCTTATTGCTAATGCA

TTTAACAATGCTTTGACAGCTATTCAGAATGGGTTTGATGCAACCAATTCTGCTTTAGTT

AAAATTCAGTCTGTTGTTAATGCAAATGCTGAAGCTCTTAATAAGTTATTGCAACAACTA

TCTAATAGATTTGGAGCCATAAGTGCTTCTTTACAAGAAATTCTATCTAGGCTCGATGCT

CTTGAAGCACAAGCCCAGATAGATAGACTTATTAATGGTCGTCTTACCGCCCTTAATGCT

TATGTTTCTCAACAGCTTAGTGATTCTACACTAGTAAAATTTAGTGCAGCACAAGCTATG

GAGAAGGTTAATGAGTGTGTTAAAAGCCAATCATCTAGGATAAATTTTTGCGGTAATGGT

AATCATATTATATCATTAGTGCAGAATGCTCCTTATGGTTTGTATTTTATCCACTTTAAC

TATGTCCCCACCAAGTATGTCACAGCAAAGGTTAGTCCCGGTCTGTGCATTGCAGGTGAT

AGAGGTATAGCTCCTAAGAGTGGTTATTTTATTAATGTAAATAATACTTGGATGTTCACT

GGTAGTGGTTATTACTACCCTGAACCCATAACTGGAAATAATGTTGTTGTTATGAGCACC

TGTGCTGTTAATTATACTAAAGCACCGGATGTAATGCTGAACACTTCAGTACCCAACCTC

CCTGATTTTAAGGAAGAGTTGGATCAATGGTTTAAAAACCAAACATCAGTGGCACCAGAT

CTGTCACTTGATTATATAAATGTTACATTCTTGGACCTACAAGATGAAATGAATAGGTTA

CAGGAGGCAATAAAAGTCTTAAATCAGAGCTACATTAATCTCAAGGACATTGGTACATAT

GAATATTATGTAAAATGGCCTTGGTATGTATGGCTCTTAATAGGCCTTGCTTCTGTTGCT

ATGCTTGTTTTACTATTCTTCATATGCTGTTGTACAGGATGTGGGACTAGTTGTTTTAAG

AAATGTGGTGGTTGTTGTGATGATTATACTGGACACCAGGAGTTAGTAATTAAAACCTCA

CATGACGACTAA

>Seq11 [organism=Hunnivirus] [isolate=Hunnivirus 1-2023-Gansu]

CAGATCCAAAAAGTACTTGATTGGCTTGGAATTTGGAAACAACAGGAAGAAGATGCCTCT

GAAGAGAAGTTTAGAGAGAAGATGAAATTGTACCCCAAGATGATGGAAAAGTATGAACAA

TACAAAAATTCTCCCAGGCACAACTGCTGGACAGAGTGCAAGAAGTGGTTTGATGACATG

CGCCGGCTGGCCGTGCTGCATGATCCCAAACTTGTTAACCTTTTCCCTAACATG

>Seq12 [organism=Bovine rhinitis B virus] [isolate=Bovine rhinitis B virus 1-2022-Ningxia]

GGCATAATACCTGTGGCAGCCAAAGCTGGTTATTCTGGGTTTTGTACCACTAGTCCTATT

ACTGCTGACCCTGTCTACGGCAAAGTAGTGAACCCACCCAGAAGACACATCCCTGGGCGC

TTCACAAATTTCTTGGATGTTGCTGATGCTTGTCCAACAATGGCCAGATTTACATCAAAG

CCCAGTATAACAACCGTTTCTGGAGCTAGTGAAAAACTCTTGGCCACGATTGACGTGAGT

CTAGTTGCTCACGAGTTATCTTTCACTTACTTGGCTGGACTTTCCTCTCTTTACGCCCAG

TATAGGGGTAGTATCAACATGCACTGCATCTACACTGGCTTTGTTAGTGACAAAGCCAAG

TTCCTGTTAGTGTATGTGCCGCCTGGAGCTGATACCCCGGCGACCCTTAGTGAAGCACAA

CATTGTATCACACTTGAGTGGGATACGGGCCTCAATTCTGAAACGGTGTTCAATATACCC

TACATTTCCCAGACTTACTACACCTCCACTCATTCCAGTGAGGCCGACATTGGCAACGTA

TCTGGACGGGTACAAGTTTTCCAAGTCACTGCCCCCTCAAGCACATCAGAACTCATCGTC

TTGTTTTCATCAGGTAGCGACTTTCAACTGCGCTGCCCTGTCGAACCTGTCAAACAAGTT

ACTGACGTTGGGGAATCTGGACACTACAAAACTTTAGACGCCACTCAACAACACGGCGAA

AAAGCCAGCACTTTCAGGCTACACACAGACGTTACGTTTGTGCTCGACAGGTACGCCATG

CTGGACGTTGTCATGGGCAACAGGATTGACTCTCACCAGAGGTACGTTAACTTGGACCCT

ACAAAGCTACCAGCCAACACTCTCATAAAGAAACTAGTTGATTCTTGTACCTACTACTTT

GCTGATCTGGAAGTGACGATTAATGCCAAGGGTACTGTTCCTAGCTGGGCGAATGTCACC

TGGTATCCCGTTGGTGCACCACGCACCTTTACTAACAACAACTTGCCAGACGAGGACAAC

GACTTCATGACCACTAGCTCAAATGTTAGTGTTGGATTTACTGGTCCTTCTAGTGGAGGT

ACAATAGCTACCTTTGCTGTTCCATACACTTCGTTTTACCGTGTGCTCCCCACCAGGTAC

GCCGGTAGGACTATTTTCACAAAACAGGAACAGTTTGAACCCTTCAACCACGGCGGTTTT

GGTGAGATAACCATTGCCGGCTCTGAAAACACTAGACACAGAGTGCTCATTAGAATGAAG

AGGACTGAAATGTACTGTCCCAGACCTCTTTACCCTAGTGCCTCTGTCACCCAACAAGGC

ACCAGACGTAAGTTCAGACTCACCAGTGAGATTGTCAAACAAGGAGCTACAAACTTTGAT

CTCCTTAAACAGGCTGGTGACGTCGAGAGCAATCCTGGTCCCACAGCTTTTAGCAAACTG

ATTGATGACTTTGGTAGTTTGTCTAACTCCATGGAGGAGATCGCCAGACACATTGGTGAT

TTCAAGGTCATGATGAAGGGTGCCGGTCCTTGGTACAAGGCTTTCAAGTACTTGTGGAAG

ACTGCCACTGTCATCGTGGCTATAACTAGAACAAAAGACGCAGTTCTTGTCGGCATGCTC

CTTGCCGATATAGGACTAGAGGTTTTTGACACTCGGATCATGATGGACAGCCTTGTGGAC

AGATTTAAGCCTTACTTCCACGTCAACCCACCAAAGTTTGACTTCAAAACTGAAGTCCTC

GACAAAGTCAGGGACTTCTTTGCCACTGACGAAGACGAAGAAGAATTTGATGACACTAAT

CCTTTTAAACAAATTTCTTTGAAGAATGTCAATGATGTCTTCAACCTTGTTAAGAACGGC

CAGTGGCTTATGAGTTTCTTCCTCTCTCTTAGAGACTGGTTCAGGACTTGGTTGGAGAGC

GAGGAGAAGTTCATCACTTACCACGACCTTGTCCCCCAGATCATTGAACACCAAGAAAAG

CTCCTGATCCCAGATGAGTACGCAGAGGCTCACAACTGGCTGGAGCGTAAGAGAGAAGTT

CTTTTACAAGCCAACCAGTATGCTCTTGCAAAACTTTGTGAACCTAAAGTTGGGCCACCA

CCTGAGACTAGGCCCGAACCAGTGGTTGTTCTGTTTAGAGGCGACTCTGGACAGGGCAAG

AGCTTTCTGTCGAACTTGATTGCCCAGGCTCTATCCAAACTTCTCACTGGAAGAGTGGAC

TCAATTTGGTCTTGCCCTCCAGACCCTGACCATTTCGATGGGTACAGAGGCCAGAAAATC

GTCATTATGGATGACCTCGGACAGAATCCTGACGGCACAGATTTCAAGTACTTTGCCCAG

ATGGTCTCCACTACGGCTTTCATACCACCTATGGCGGCCTTGGAGGACAAAGGTAAGGTA

TTCAACTCACCAGTTATCATTGCAACTACTAACATGCATGAACACTTCACGCCCAAAACC

ATGGCGTGTCCCGGCGCACTTCTTCGCCGTTTCACCTTTGACTACGTTCTTGCGGCCAAG

AAACCCTACATCAGAGAAAAGACTGAGACACTCAATGTCAGGAAAGCCTTGGCAGCCAGT

GGGGAAGAGTGTCCTTGCGGACTCTTTGAGTTCGACTGCCCTCTTCTCAACGG

>Seq13 [organism=Bovine rhinitis B virus] [isolate=Bovine rhinitis B virus 2-2022-Ningxia]

CAATGCATAACTCTTGAGTGGGACTCTGGACTTAACTCAGAGGCTGTATTTACTATACCT

TACATCTCCCAGACCTATTACACCTCAACTTACTCAGGTGAGGCAGATATAGGTAATGTT

TCTGGGTGGATACAGATATACCAGGTGACAAAAGCTAAAGCTAACAACCACCTTCTTGTC

ATGTTTTCGTCTGGTACCGATTTCCAGCTCAGATGCCCCATTGAACCTGTCAGGCAAGTG

ACTGACGTTGGCGAGTCTGGCAACTACAAGACCATTGATGCACGCCAACAGCAAGGGCAA

CACTCGCCCACGTTCCGGCTTCACACCGATGTCGCATTTGCCTTGGATAGATACACCCAG

ATAAAAAACATTGGGGCCAACAGGACAGATAGCCACAGAAATATCACCAACTTGGATTTA

ATGTCTCTGCCAAACGGGACATTTGTCAAGACTTTGCTTGCTTCATGCACTTACTACTTC

TCTGACTTAGAAGTTGCTGTGAACTCTAGGGGCACTGTACCTGCTTATGCCCACGTCACT

TGGTACCCTGTTGCCTCTCCTAAGTCTTTTACCCAAAATCTTTACACTGACATAGACAAT

GACTTCATGACTATCAATTCTACAGTTAGTGTTGGTTTCGAAGGGCCTAGTGGTGGCTCT

AGTGTTGCTGCCTTTGCCATACCCTACACCTCAATTTACCGAGTTTTGCCCACTACTTAC

AACGGCAGCACCTTCCACGGTACCGGTGGAAAGGGTAGGAAATTTAACCACACTGGTTAT

GGTGAAGTCTTTATAACTGGTCTTGAGAATGTCAACCATAGAGTCATGGTCAGGATGAAA

AGAACTGAGATGTACTGTCCCAGGCGAATACACACTCAACTGGGTGGTGCCACGCAAAGT

ACCAGACACAAAATGACTCTTGTTAGTTCTATAAACAAAGAGGGGGCTACCAACTTCAGT

CTTCTTAAGCGGGCTGGAGATATCGAGAGCAACCCTGGCCCCACAGCTTTCAGCAAACTG

ATTGACGACTTTGGTTGTCTGTCCAACTCTATGGAGGAAATCGCCAGACACATCGGTGAT

TTCAAGGCCATGATGAAGGGTGCTGGACCTTGGTATAAAGCTTTCAAGTACCTTTGGAAG

ATTGCTACTGTCATCGTGGCAATAACCAGAACAAAAGACACAGTTCTCGTCAGCATGCTT

CTTGCCGACATAGGGTTGGAAGTTTTTGATACTCGAGTCATGATGGACAGCCTCGTGGAC

AGGTTCAAACCATACTTCCACGTAAACCCACCAAAGTTCGATTTCAAGACCGAAGTCTTG

GATAAGGTCAGAGACTTCTTCGCCACTGAAGACGACGAGGAGGAATTTGATGACACCAAC

CCCTTTAAACAAATCTCTCTGAAGAACGTCAATGACGTTTTCAATCTCGTGAAGAACGGT

CAGTGGTTGATGAGTTTCTTCCTCTCTCTAAGGGACTGGTTTAGGACCTGGCTGGAGAGT

GAGGAGAAATTTATTACCTACCACGACCTTGTCCCTCAGATCATTGAACACCAAGAAAGG

CTTTTAGTTCCAGACGAGTACGCAGAAGCCCACAACTGGCTTGAACGTAAGAGAGAAGTC

CTTTTACAAGCCAACCAGTACGCTCTTGCCAAACTCTGTGAACCCAAAGTTGGACCACCG

CCTGAGACTAGGCCTGAACCAGTAGTTATTCTGTTTAGAGGCGACTCTGGACAGGGCAAG

AGTTTTCTTTCAAATCTGATTGCTCAGGCTCTTTCCAAACTTCTCACTGGAAGGGTTGAC

TCAATTTGGTCTTGTCCTCCAGACCCTGACCATTTTGATGGATACAGAGGTCAGAAAGTC

GTCATCATGGACGATCTTGGGCAAAATCCTGATGGTACTGACTTCAAGTACTTCGCCCAG

ATGGTCTCCACCACAGCCTTTATACCACCGATGGCAGCCTTAGAAGACAAAGGTAAAGTG

TTCAATTCACCAGTGATTATTGCCACCACCAACATGCATGAACACTTCACGCCTAAGACC

ATGGCGTGTCCTGGTGCACTTCTCCGCCGCTTTACCTTTGACTATGTTCTTGCGGCTAAG

AAACCCTACATTAGAGAAAAGACTGAGACGCTCAACGTCAGGAA

>Seq14 [organism=Enterovirus] [isolate=Enterovirus-2023-Shannxi]

CATGGAGTGATAGGTATTATCACAGCAGGTGGCCCAGGTCTTGTTGCT

TTCGCAGATCTACGCGACCTGTTCTGGGTTGAGTACGAGGTCATGGAACAGGGTGTGGCT

GACTACATTCGCAGCCTTGGCAATAGCTTTGGTCAGGGCTTCACCGATGAAATTTCCAGG

CTTGCAGAGCAGTTCAAAGACATGATGACCGGCGCAGATGGCTTGGTGGAGCAGTGTATA

AAGACCTTCGTCAAGGTGGTTTCAGCAGCGGTTATTGCAACTAGGGCAGAGGGTGATGTG

CCGACCATCCTTGCAACCTTAGCTCTTATAGGGTGTGACACCAGCCCCTGGAGGTGGCTA

AAGAAACAATTTTGCTCCATTTTCAAACTTCCTTATGTTGAGAAACAAGGTGATGACTGG

GTTCGCAAGTTTACCACCTATGTGAACGCCTTCAAGGGTCTGGATTGGGTCGGAGAGAAG

ATAATGAAATTCATTGAGTGGCTTAAAAATGTGCTTCTGCCACAAGCTAAAGATAGAGTT

GAGTTTGTCAACAACCTGAAGTCGCTGCCTTTGCTGGAAGCCCAGATAGCGACTCTAGAA

CACTCTTGTCCGTCCACCGAACAGCAGGAGGTTCTTTTTGGAAATGTCCAATACTTGGCC

CACCACTGTCGCCGGTATGCACCCCTCTACGCAGCGGAGGCAAAGCGCGTATACGCGCTG

GAAAAGAGGGTGCTAGGATTCATACAGTTCAAGAACAAGCAACGAATTGAGCCCGTATGC

CTCCTAATACATGGCACTGCTGGCACAGGCAAATCATTGGCCACTTCCATTATTGGTCGG

AAACTGGCCGAATATGAGCACTCTGAAGTGTATGCTATCCCTCCAGACAGTGATCATTTT

GATGGGTACCAACAGCAAGCAGTGGTGGTGATGG

>Seq15 [organism=Enterovirus] [isolate=Enterovirus-2022-Ningxia]

TTTTGGATTGAGCATGAGGCCATGGAACAGGGCATCACAGATTATATTAGAGATCT

TGGCAATAGCTTTGGCCAAGGTTTCACAGATGAGATCTCTAAATTTACTGAGCAGCTCAG

GGAACTCATGACTGGTGCCGATGGCCTAGTGGAACTCTGCATAAAAACCTTTGTGAAAGT

GGTATCAGCCATAGTTATTGCTACCAGAGCTGAGGGAGATGTACCCACAATCCTTGCGAC

CCTCGCCCTCATTGGGTGTGACACCAGCCCTTGGAGATGGCTTAAGAAGCAGTTTTGCAG

TATTTTCAAGATTCCATACGTGGAAAAGCAGGGAGATGATTGGGTCAGAAAGTTCACCTC

TTACATCAATGCCTTTAAGGGCCTTGATTGGGTTGGAGAGAAGATCATGAAATTTATCGA

TTGGCTTAAGAATAACTTGATTCCCCAGGCAAAAGAGAAGGTTGAATTCACCACTAACCT

AAAGTCCCTCCCGCTTCTTGAGGCTCAGATTGCCACCTTGGAACACGCATGTCCTACCAC

AGAGCAGCAGGAAACTTTATTCGGTAACGTGCAGTATTTGGCCCACCATTGTCGCAGATA

TGCTCCATTGTATGCTGCAGAAGCAAAGAGGGTGTATGCCCTAGAGAAGAGAGTCTTAGG

TTACATACAGTTCAAGAATAAGCAACGAATTGAACCTGTATGTCTCCTGATCCACGGGAC

TGCGGGGACGGGAAAATCCCTTGCCACATCCATCATTGGCAGAAAACTTGCAGAATATGA

ACACTCCGAGGTTTATGCCATCCCACCAGATAGTGACCACTTTGATGGGTATCAGCAGCA

GGCTGTGGTCGTGATGGATGATCTGAATCAGAACCCAGACGGGAAGGACATGGTTGCATT

CTGCCAAATGGTTTCAACGGTGCCTTATCACGTACCAATGGCCGCGCTGGAGGAGAAAGG

GATGCTGTTCACCAGTGCATATGTTTTGGCCTCGACCAACAGCGGGTCCATTCACCCACC

AACCGTTTCCAACTCCAAGGCCCTTTCCAGAAGGTTTGCTTTTGATGTGGACATAGAAGT

CTCCCAGAATTACAAGACAGAGAGTAACACCCTGGATGTGGTTAAGGCCACCCAAGCATG

TGATGATTGTTGCCCAGCCAACTTCAAGAAGTGCATGCCACTGGTGTGTGGGGAAGCCTA

CACCTTAGTGGATAGAAGGACCAAGATCAGGTATTCCATTGATCAGATGATTTCAGAGAT

GAGGCGAGAGTGGCAGCGCCGCAACAGAGTGGGCTCAGTGATTGAGGCTCTTTTCCAGGG

GCCCCCCCAATTTAAACCGCTTAGAATTACTGTTGACCCCGAAGTACCACCTCCACCAGC

AATAGCTGATCTTCTTGCTAGTGTCGACTCTGAACAAGTCAGGGAGTATTGCAAGCAGAA

AGGGTGGATTGTGGAAGTTCCAGTTACAGCCATGACCTTAGAGAAAAATGTGAGCATTGC

TGCTACAGTCATGTCCAGCTTGATCTTATTGACCTCAGTTATTACCTTAGTTTATCTAGT

GTATAGGTTGTTTGCTGGCTATCAGGGCCCCTACACCGGTCTTCCAAATAAAAAGCCCAA

AGCCCCTGTTCTTAGAGAGGTCAAAGCGCAAGGGCCCTTAATGGACTTTGGTGTTAGCAT

GATGAAAAAGAACATTGTCACCGTGCGGACTGGATACGGAGAGTTCACAGGTCTAGGTGT

GTATGACACAGTGCTGGTTCTCCCAAGACACGCACACCCAGCAGAACAAATGATGGTCGA

TGGGGTGGAAACACCAGTCCTAGACGCATACAACCTAACAGATGAAGAGGGCGTCTCCCT

GGAGCTCTGCCTAGTCACCCTGAAGAGAAATGAAAAATTCAGAGATATCAGAGCAATGAT

CCCAGAGAACCCAAGCGGAACCAATGAGGCTGTGGTGTGCGTGAACACCAGTGCATTTCC

AAATGCCTTTTTGCCCGTGGGCAAGACCGAGTACTACGGCTACCTCAATCTAGCCGGGAA

TCCCAC

>Seq16 [organism=Nodaviridae] [isolate=Nodavirida-2022-Ningxia]

GCCACAGGGCTGAGCTTTTTGCTCCGCTTGTCTTATGAGCTGTTACCTAAATTTAGTTAT

AACAGCTGGAAGCACCTGAGGGATGCCTTCCGTCGTGACGAATTGCCCGTTTACCAAGTC

GGACCTAGTGCCCATTCTCACCCGAATGCGGCTGCCGACCGGAGCCGGTGCTCCCAGTGG

ATTGATGACTTCATTGTACGGTTTGGGCTTGAACCGTACTCGATTTCCATGTCAAACCGA

GATATTGACAATGGACTTGCTGGGCAGAGGTACCGCTTTGTAGCTAAAGATTACGCGCTA

CCGGTTGTAGACGATCCCGTCTCCCATCGCGATGTGCTCAAATTTATCGACGTGGACTAT

TACGTCGAACCCAGCCAAATCTTCAATCCCCTTCGCCCCGTGTTAATTTACACCTTTCAT

GCCATCAAACCGTGCGGCAGAATTCCAGATGGCTACTACACGTCCAACGGCAACGTGTTG

ACTGTTACTTACGGGGGTGGAGCTACCTACAAGCATCAAGTGTGGAACTACTTTACGGAT

CATGTGGTTTACCACCATTGGTACGGGACATCCGTGTGGTCAGTGGACACAAGGCCCACA

GATGATCGTCACATTTTAGTGCTCCTCACGCCTATCGCCCTTGTGCCATTCAAGTGGCTT

GACGGGCCTGAGTTCGAGCGCAAGCAGTATCACGGTGACTTTCAATGGACTCGGTCTTAC

CGTGCTGCGGACGCTTATTATCACATGGCTTATGCTGGCTCCACATGCTGTGTGACAGTC

GCTGAGGCAACCCTTCGCGGCATGCTCGTCAGATATGACCAGGCTAAGATGCCGTCACTA

GCTGACTTTGAGCGCGTCCTTAACCATACTGCGGACAAGCGTGCTGTTGTTAACGCTCCG

TTTCTGTTCCAAAACGTTGAGCTCGTGAGGAAAATGTTGAATCAGGCCAGGATGATGCCG

GTCGAGATTGATCCGTTAAATTATCAAGCGCCTGGTCCTCTGGTTTATGAGGACGCCGCA

CCCACAGCAAACATGGCATGTCCACCATTAGCTGATGGCGCTGTTGCTCCCGGTAAGTCG

TACAATAATGATGTACAATGTGTCCAAGAACGCCTCACGAAGATTAACAACACTAAGCAG

CCTCCTCCTAGAGTTCAAAAGTGGGTTAAGGAGTTTATTTATTCGCTGGTCCCTTCCCAC

ATTCGTGGCACAATCCGGCCTTGGAACATTGAATCGGTGTGCGATCAACAGAATCGCCCC

ACCCAGCGCGCTAGCTGGCAGCGCGTACTTAACTGGGTACCTTTCATTAAATTCGAGGTG

CAATCATTTCAGAAGGCCGAGACTTACCCCGACGTTAAAGCTCCCAGGAATATTTCAACC

ACGCCCGCCAGCCACCGAACACTCTACGGCTCTTACATTTATGCCATTAGCGAACACGTC

CTCAAGCCTCAACCGTGGTACGCTTTTGGCCGCAATTTAGCCGAAATTGCCCATCGGGTT

CATCAGGTTTGCCGTCAATCACAATTCGTAGTGCCTACAGACTTCTCCGCATTCGATGGC

ACCCACTCGGAGTACATGGTCTCCGTGGAAGAGCTTCTTGGAGAACTTCTGTTTCACCCC

TCGTGCCTACGAGAG

>Seq17 [organism=Nodaviridae] [isolate=Nodavirida-2023-Shannxi]

GCCACAGGGCTGAGCTTTTTGCTCCGCTTGTCTTATGAGCTGTTGCCTAAATTTAGCTAC

AACAGCTGGAAGCATCTGCGTGATGCCTTCCGTCGTGACGACTTGCCCGTTTACCAAGTC

GGACCTAGTGCCCATTCTCATCCGAATGCGGCTGCCGACCGGAGCCGGTGCTCCCAATGG

ATTGATGACTTTATTGTGCGGTTTGGGCTTGAACCGTACTCTATTTCCATGTCGAACCGA

GATATCGACAATGGACTCGCTGGGCAGAGGTACCGCTTTGTAGCCAAAGATTATGCGCTA

CCGGTTGTAGACGACCCCGTCTCCCATCGCGATGTGCTCAAGTTTATCGACGTTGATTAT

TACGTCGAACCCAGCCAGATATTCAATCCACTTCGCCCCGTCTTAATTTACACCTTTCAT

GCCATCAAACCGTGCGGCAGAATTCCTGATGGCTACTACACGTCAAACGGCAACGTGTTG

ACTGTGACTTACGGGGGTGGAGCAACCTATAAACATCAAGTGTGGAACTACTTTACGGAT

CACGTAGTTTACCACCATTGGTACGGGACATCCGTGTGGTCAGTGGACACGAGGCCCACG

GATGATCGTCACATCTTAGTGCTCCTCACGCCTATCGCCCTTGTGCCATTCAAGTGGCTT

GACGGGCCTGAGTTTGAGCGCAAGCAGTATCACGGTGACTTTCAGTGGACTCGGTCATAC

CGTGCTGCGGACGCATATTACCACATGGCTTATTCTGGCTCCACTTGCTGTGTGACCGTC

GCCGAGGCAACCCTCCGCGGCATGCTCGTCAGGTACGACCAGGCTAAGATGCCGTCGCTT

GCTGACTTCGAGCGCGTCCTTAATCATACTGCGGACAAGCGTGCTGTTGTTAACGCTCCG

TTTCTGTTCCAAAACGTTGAGCTCGTGAGGAAAATGTT

>Seq18 [organism=Circovirus] [isolate=Goat circovirus 1-2023-Shannxi]

GGGCATGAAGTAGGCGAGAATGGAACTCCGCACTTGCAGGGTTATATAACATTCTCTACT

CGTAAGTCATTTAACACCATGAAAAGACTCATAGGAGATAGAGCTCATATAGAAGTAGCA

AGAGGAAAAGTCCAGGACAATATAGACTATGTTACAAAGCAGGATCACGATCACTACTTT

GTAAAAGGAGAAGCACCAGCAGAAAGCTCAGAAAGAGGAGCAGAAGCAACCAAAAGAAAA

TGGGAAGAAGCAAGAAAGGCAGCAAAAGAAGGAAGATTTGAAGATATACCCTCAGACTTA

TGGATACGATACAGAAACAGCTGGAAAAACGAATACCAAGAAGAAGTAAACAGGAATGTG

ACAGAAATCAAAGACTTCAACTTAAAAGAACACTTCTATTGGATTTACGGTCCAACGGGG

ACCGGTAAATCACACTTAGCTAGAGCTTTAGCTAAACGATTAGATCCTGACAATGAACCA

TATCTAAAATCAATAAATAAATGGTGGGGCGGATTCAAAGGACAAAAAGTAACCTTAATA

GAAGAAGCATCTCCAGAATCAGTAAAATTCCTAGCAAATCTCTTCAAACAATGGTGCGAT

AAATGGCCGTTTCCAGCAGAAGTAAAAGGCGGAGCATTTGAAAATGGAATTCGACCAGAT

TATATCATAATCACGTCGAATTACTCCATAATGGAATGCTTCCCAAACGAAGAAGATTAT

CTACCAATGAAAAGAAGGTGCACAGAATTCTACAAACAAACTAGAGAATCATGGTTCGAT

GTTTCAGAACCAGAGCATGATACACAACAACTAAGTCCACTAACGCAAGATATACCAAAA

CTACCGAGAAACGAGTCTTACGAAGTAATCGTAGACTCAGAAACCATAAATTCATTTACT

AATCCGTAA

>Seq19 [organism=Circovirus] [isolate=Goat circovirus 2-2023-Shannxi]

AGGTGATAAATGTCATATTGAAGCAGCAAAAGGTTCAATCAAAGAAAACATCGCATATTG

TAGTAAAGATGGGGATGTTTTCATTAAAGGAGTTGAACCAGAAGAACAAAGTGAAAAAGG

GGGTCAAGCAACAAAAAGAAAATGGGAAGACGCACAAAAAGCAGCAAAAGAAGGTAGATT

CGAAGATATACCAGCCGACTTATGGATAAGATATCGAAATTCATTCAAACAAGAATATCA

AGAACAAAGAGTAGAATCAGTCAAACCAATAGAAGGTGATCTAAAAAAACACTTCTTTTG

GATTTGGGGACCAACAGGTACTGGCAAGTCATGGTTAGCAAGAGAAATTGCTAAAAAAAT

AGCACCAAATGAAGAACCTTTTCTCAAACAACTAAACAAGTGGTGGTCAGGATACAGAAT

GCAAAAAGTAGTAATCATTGAAGAAGTAAACCCTTCAATATGTCAAATAATGTCAAGTTA

CTTCAAACAATGGTGTGATAAATGGCCATTCTCAGCAGAGACTAAAGGAGGTGCATTCGA

AAGAGGTATAAGACCTGAATACGTCATTATTACGTCAAACTATTCAATCGATGACTGTTT

TGAAAAAGAAGAAGATAGACAACCAATGCAAAGAAGATGTAGAGAAGTATTCAAAGAGTC

TAGAGATGCATTCGTGTTCTTCCCAATTGACACACAGTCTCAATCAGTCGAACAAGAGAC

GCAAACTTTACAAGTTCGCGATCCACCAGGTAACCAAGAAGCCAACAGCCAAATATCACA

ACAAGTACCAAAAGAATGGTTAGAAGAGTGTATGTCACAACAGTTCTAA

>Seq20 [organism=Circovirus] [isolate=Goat circovirus 3-2023-Shannxi]

ATGGCAAGGGCAAAACGTTGGGTCTTTACCTTGAACAATTATACAACAGAC

GAAGTCGAACAACTTAAACAAGTAGATTGCGAATACCTAGTATTCGGTTATGAAACAGCA

CCAGACACTGGGACACCACACTTACAGGGTTACATAACATTCACTAATAGGAAGACAATG

ACAACTCTGAAAAGATTACTAAACGATAGATACTACTTTTCAGTAAGTAGAGGTTCAGTC

CAAGAAGCATCAGACTATTGCAAAAAAGGAGGTAATTACTTCGAAAAGGGAACTCCACCA

GCAGAAATAGGACAAAGAGGAGGACAAGCAACCAAAAGAAAATGGGAAGAAGCACTAATA

GCAGCAAGAGAAGGAAGATTCGATGACATTCCACCAGATCTATGGATAAGATACAGAAAT

TCATGGAAACAAGAATATCAAGAAGAAGTAAACAAAACAACAGTAGAAATAAGAGATTTC

GACCTGAAGAACCATTTCATTTGGATTTATGGTCCTACAGGAACAGGAAAATCTCATTTA

GCTAGAAGTCTAGCCAAATCAATTACAGATGAACAACCATACTTAAAAGGACTAAATAAA

TGGTGGAACGGCTACAAAGGACAAAAAGTAGTCATCATAGAAGAAGCGACACCAGATAGT

TGCAAATACCTAGCAAGCTTATTCAAGCAATGGTGTGACAAATGGCCATTTACCGCCGAG

GTAAAAGGAGGATCCTTTGACCACGGCATAAGACCAGATTATATCATCATAACATCAAAC

TATAGCATTGAACAATGCTTTCCAGATGAAAATGACTATCAACCAATGAAAAGAAGATGT

CATGAATTCGAAAAAAAAACAAGAGAGAGTGTGCTGAGTATAGAACCTGAAAGAGACACA

CAAGTCTTACCTCCAGAGCCAGTTCCTCTGGCTACTCTCCCAAGGGCAGAGTCCGTAGCA

TCAATACAAATAGATTCACCA

>Seq21 [organism=Circovirus] [isolate=Goat circovirus 3-2023-Shannxi]

AAAAATGGCCAGAGCAAAACGTTGGGTTTTTACATTAAACAATTACACAGAAGAAGAAGT

TGAACACTTGAAACAAGTAGATTGTGAATACTTAGTATTCGGATATGAAACAGCACCAGA

TACGGGTACTCCACACTTACAAGGATACATAACGTTCAAAAATAGGAAAACCATGACGAC

CTTAAAAAGACTACTAAATGACAGATACTACTTTTCAGTAAGTAGAGGATCAGTATCAGA

AGCATCAGAATATTGCAAAAAAGAAAACAACTATTTCGAAAAAGGAACACCACCAGAAGA

AACATCAACAAGAGGAGGTCAAGCAACAAAAAGAAAATGGGAAGAAGCAATAAAAGCAGC

AAGAGAAGGAAGATTCGAAGATATTGCACCAGACCTATGGATAAGATATAGAAATTCATG

GAAACAAGAATATCAAGAAGAAGTAAACAAATCAACAACAGAAATAAGAGACTTTGACCT

TAAAAACCATTTTATATGGATATGGGGTCCAACAGGAACAGGAAAATCACATTTAGCAAG

AAGTCTTGCTAAATCCATTACTGAAGAACAACCATACCTTAAAGGTCTAAACAAATGGTG

GAATGGATACAAAGGACAAAAAGTAGTAATCATAGAAGAAGCTACACCAGATAGCTGTAA

ATATTTAGCAAGTCTATTTAAACAATGGTG

>Seq22 [organism=Circovirus] [isolate=Goat circovirus 1-2022-Ningxia]

ATGGCAGCTAGTCCAAACAGCCGAGCAAAAAGATGGTGCTTTACACT

AAACAACTACACAGCACAAGAAGAAGATAATATACAAAAGACCGAATGCCAATATCTAAT

ATATGGCCACGAAGTCGGGGAGAATGGAACTCCACATCTCCAAGGCTATGTGACGTTCTC

CACACGTAAATCATTTCGCACCATGAAAAAGCTACTTGGAGAAAGAGCTCATATAGAAGT

AGCGAGAGGAAAAGTTCAAGACAACATAGACTATTGTACAAAAGAAGACCACAGCAACTT

CTTCATGAAAGGAGAGGCACCAGCAGAATCATCAGAAAAAGGTGCAGAAGCAACAAAAAG

AAAATGGGAAGAAGCAAGAGAAGCCGCGAAAAAAGGAAGATTTGACGAAATTCCATCAGA

TTTATGGATAAGATACAGAAACAGTTGGAAACAAGAATACCAAGAAGAAGTAAACAATAA

TGTCACAGAAATAAGAGACTTCAACTTAAAGGATCATTTTTTCTGGATTTACGGACCAAC

AGGAACCGGTAAATCACATTTAGCAAGAGCACTTGCTAAAAAAATAGATCCTGATAATCA

ACCATATCTCAAACCATTGAACAAATGGTGGAATGGATTTAAAGGACAAAAGGTGACATT

AATTGAAGAAGCAACACCAGATGCTTGTAAATTTTTAGCAAATTTATTCAAGCAATGGTG

CGACAAATGGCCATTCACTGCAGAAGTGAAAGGTGGCTCATTTGATAATGGAATTCGTCC

AGAATATGTCATAATCACGTCAAACTATTCAATCATGGAATGTTTCCCTAATGAAGAAGA

CTATAAACCAATGCAAAGAAGATGCACAGAATTCTTCAAAGATAAAAGAGAAGCATGGTT

CACAATACCAGAAGAAGAACACGATACACAACAATTACCTCCTAGTAGTCAAGACGTACC

AGTACTTCCAAGGACAGAGTCCTATGAAATCTTAGTGGACGAAAATACATGTTCGTACAA

TTATCCGTAA

>Seq23 [organism=Circovirus] [isolate=Goat circovirus 2-2022-Ningxia]

ATGACGTCAAAAAAACACAAAAAATTAAATCTGAAATGGTTTCATTTCAAAAAAAATGTC

ACGTGGCAAAAGGTGGTGCTTTACCATTAACAATTATACAATCGAGGATATCAAAAGACT

CGAACAAATAGACTTCGAATATATAGTCTATGGAAAAGAAAGTGGAGAACAAGAACATAC

TCCTCATTTACAAGGTTTTGTAATTACAAAACAAAGGAAAACTTTCAATGCAATCAAAAA

ATTAATAGGTGATAATGCACATATCGAAATAGCAAGAGGAACAAACAAAGAAGCAAGTGA

TTATTGCAAAAAAGAAGGTGATTATGCGGAATTTGGTACATTGCCAAAAGAAAGAGGAAG

ACAAGGAGGAGAAGCAACCAAAAGAAAATGGGAAGAAGCATATAATGCAGCCAAAGAAGG

AGACTTCGATTCAATTCCAGCGGAATTATATATCAGGTATCGAACCTCCTTCAAAGCAAT

ATATCAAGAAGAAGTTAATAGAAACACGTCAGCAATAGGTGACTGGGACCTTAAACATCA

CTTTATATGGATATATGGGCCAACAGGAACAGGTAAATCACATATGGCAAGATCAATTGC

AATGACAGTAGATCCGGAAAAACCGCCATACTTAAAAGGATTAAATAAATGGTGGTCAGG

TTATCAAATGCAACGATGCGTAATAATAGAAGAAGCAAGTCCAGAAGCATGCAAATATCT

AGCACCTTTATTCAAGCAATGGTGTGATAAATGGCCATTCACAGCAGAAACTAAAGGTGG

AAGTTTCGAACATGGAATAAGACCAGAATTCATAATAATAACTAGCAATTACTCAATAGC

AGAATGCTTTCCAGAAATCAATGATCAAGAACCAATGCGCAGAAGATGTTTCGAGTTCTA

TAAAGAAACTCGTGAAAGTTGGCTACCTTTCGACTTCAATGAATTTAATGATGCTACTCA

AATGTTACC

>Seq24 [organism=Circovirus] [isolate=Goat circovirus 3-2022-Ningxia]

AACAACAGAGCAAAAAGATGGTGTTTTACCCTTAACAATTACACCAATGAAGAGGAGCAA

AAGATCCAAGAAGCAAATTGTGAATTTTTAATATATGGGCATGAAAGAGGCGAGAATGGA

ACTCCACATCTTCAAGGCTATATAACATTCACTACTCGCAAGTGTTTTAACACCATGAAA

AGATTACTAGGAGATAGAGCTCATATAGAAGTAGCAAGAGGCTCAGTAATGGCAAATATT

GACTATGCAACTAAAGAAGATAACGAACATTATTTTATAAAAGGTGAAGCACCAAAAGAA

GCAAGTGAAAAAGGTGCACAAGCAACAAAAAGAAAATGGGAAGAAGCAAGACAAGCAGCA

AAAGAAGGAAGATTCGAAGATATACCATCAGACTTATGGATAAGATATAGAAACAGTTGG

AAAACAGAATATCAAGAAGAAGTGAATAAAAATACAACTGAAATAAGAGATTTCAACTTA

AAAGACCATTTCTACTGGATATGGGGACCTACTGGAACAGGGAAATCACATTTAGCTAGA

GCACTAGCTAAAAAATTAGATCCAGATCACCAACCATATCTCAAATCACTAAACAAATGG

TGGAATGGATTCAGAGGACAAAAAGTAACCTTAATAGAAGAAGCAACTCCAGAAGCATGT

AAATACATATCATCAATGTTCAAACAATGGTGTGACAAATGGCCTTTTACAGCAGAAGTA

AAAGGAGGATCATTTGATTGTGGTATTCGACCTGATTATATAATAATCACATCGAATTAC

TCAATTCAACAATGCTTTCCAGCTGAAGAAGACTATCTTCCAATGCAAAGAAGATGCACA

GAATTCTATAAAGAACACAGAGAATCATGGTTTGAAATACCAGATTCTCCTGAACATGAT

ACTCAACAATTAAGTCCAAACACTCAAGATATACCGAAATTACCGAGAACCGAATCATAC

GAGATCATCGTAGACGAAGACACAAGAAACTCGATAAACTCGTTCACTAATCCGTAA

>Seq25 [organism=Circovirus] [isolate=Goat circovirus 4-2022-Ningxia]

ATGACTACTCTAAAGAGACTACTAAGTGATAGATACTATTTTTCAGTAAGTAGAGGA

TCAGTACAAGAAGCATCAGAGTATTGCAAAAAAGAAAACAACTTTTTTGAAAAAGGAACT

CCACCGGAAGAAAGTCAAACTAGAGGAGGTCAAGCAACTAAAAGAAAATGGGAAGAAGCA

ATAAAAGCAGCAAGAGAAGGAAGATTTGATGATATCCCACCAGACTTATGGATAAGATAT

AGAAATTCTTGGAAACAAGAATATCAAGAAGAAGTAAATAAGTCAACCACAGAAATAAGA

GACATAGACTTAAAAAATCACTTTATATGGATATGGGGCCCTACAGGAACAGGTAAATCC

CATTTAGCTAGAAGTCTAGCTAAATCAATTACTGAAGAACAACCTTATTTAAAAGGTTTA

AATAAATGGTGGAATGGCTACAAAGGACAAAAAGTAGTAATCATAGAAGAAGCAACACCA

GATAGCTGCAAATACCTAGCAAGTCTATTCAAACAATGGTGTGACAAATGGCCTTTTACA

GCAGAAGTAAAAGGCGGCTCATTTGATCACGGTATAAGACCTGATTATATAATTATTACA

TCCAACTATAACATTGAACAATGTTTCCCAGATGAGAATGATTACCAACCAATGAAACGT

AGATGCCACGAATTCGAAAAAAAAAATAAAGAGAGCGTTCTAAGTATCGAACCAGAGAGG

GACACACAAGTACTACCTCCAGAGCCAATTGAATTGGCAACTCTCCCAAGAGCAGAATCA

GTATCATCTATAATAGTAGATGAAGACACAATAGGATTTAGTAATTACTAA

>Seq26 [organism=Adenovirus] [isolate=Goat adenovirus 2-2023-Shannxi]

ACCTTTGCCCATCGCGTGTGTGTTTACAGTAAGGCTTATTAATGATTCACAGAGTTTTTA

TTGAGTTCTGTGGAATTTCTGTTTACACCCGTAGAACTTTAGACTCTCTGGCAATAAATG

GGTGTGCTGTGAAAACACCTAGTTGTTCTACACATGAGGTCACTTTTACGACTCTACAGG

TAAAAATGGCTCTCAATAGCTCCAAAGGTCTATTATACGTCAGTAGGTGCGGCCGGCATT

TTCTGTTTCAGTTCCTCATAGCCAGGTATAAAGCCTTCACAGGTGTTCAGAGTCGCCACT

CTTGAGTCTAAGAGAAGTAGAGCTTTCTCCTCTACACTTAGACATCATGAGGCACCTGCG

CTTAGCTTTTGACTGGAGATTCTGGGAAGCAGCAGAAAACCTTCTGCAAGACTTACACAC

ACCAGAAGACGACGATGAAGGCTTTTCAGAGCCTTTAACGCTGCAAGATTTAGTAGAGTT

AGAGTCTCCAAACGCTGTTGATTACTTTTTCCCAGACGCGGATGTTCCACTAGAGCTGCC

TACTGACGCTGCTGCTGAAGAGTTGCTGCGCTGTGATGAAGAAGTAGTAGATTTATCTAC

TTTATTCGACAGCGAGACTGAATCTCCACCTGGTTCACCATTTTCAGCTGTGGAGTTAGA

CTATCCTGAACATCCGGGCCACAACTGCTCAGCTTGTGATTATCATCGGAGAGCTACTAG

AAATGAAGAAACTTTGTGTTCCCTGTGTTATATGAGAAAAAACGCGTTTGCTGTATATGG

TAAGCGAATGTTTGCTTTACATTTAAAATGATTTAGTTTTATAAGAGTGATTTGTGAGTA

AACTTTTATGTCATTTATTTTATAGAACCTGTTTCACCTGCTGATCCAACTGTTGTTGCT

ACTGAAAATGTTGACAGTGAAGATGCAAGTTGTGATGATGACTCAGTGGAAAGTACCACA

CCTGATAATAGCCGTAAACGCCGTCATCAGGAGACTTTGCCCCTGTTTAAAAGACAACGC

CTTGATGAACCTTTGGACTTGAGTGTTCAGAAAAATCAATAAACCCTCCCTCAATAAACT

TGTGCTGACATCAATAGTAGGAGTGGTTTTCTGTATAAGTACTGGCTCAGGTGCGCGATT

TAGTTAGAGCAACAAGAGCTGCTGATATGGACTTGTCACTTCAGCTTTGCGAGCAGCTAA

GCAGCTTACCTCAGTTGCGGAGGGTGATCTACTACGCAACAAATCGGGCAAGTTGGTGGT

CTAGGACTTTCTTTGGGGGCAGACTCGCTAACTTAGTTTATAAAATTAAAGTTGAAGAGG

CAGAATCTCTCCAGGGATTGTTTCAGGAAGATGAGGGTTTTTGGCAGATGTTTGGCAGTG

GCAGAACACTAGGCTATGAGTCAAAAATTGTGCCTTGGCTAGACTTTTCAACTCCAGGGA

GAGCAACTGCTAGTTTATCCTTGCTTGCTTACATTGTAGACAGTTTGGATAAACAGTCTG

AGCTTTCTAGTGACTTTATTTTAGACGCTGTGTGTGGACCAGTATGCTACAAGCTCAAGA

TGCTGGTGTCCCTGCGGAGGCTTCAGGGGCATCTGTCAGGGTCCAGCGTGGATTCCACTT

GCCCTTCAGCAGAGGAAACGGATTAGAACCTAATAGAGTTACATACCAGCAAATATTAGG

CGAGTTTCAAGCTGATCCATTTTTCACTAATGATAGATATGATTTTGAGCATGTTGCAAC

CCACTGGTTAGCAGAGGGAGAGGATTTAGAAGAAGCAATTAAGCAGCACGTAAAGGTTGC

TTTGGATCCAAATAAAGAGTATGTAATCCACAAGCCGGTAAAAATTAAAACTTTATGCTA

TATTATAGGAAATGGAGCTAAAGTAAAAATAGCATGTAAGGAACCTTTTGGCTTTGAAGT

GTATTGTAGGTCGCCTAGTCCTGGCATAGTAGGTATGTGGGCAGTGACTTTTCACAATAT

AGTCTTTGAAAGGGATAGGACTATGCCAGGTGGGGTTGTAAATTCTAGAACTTTTGTCCT

ATTTCATGGCTGTAACTTTCTAGGCTGTATGGGAACTGCTTTAAGTTTTAGCTGCGGAAG

TGAAATCAGGGGGTGTCATTTTTTTGCTTGCTACAAATGCATAGATAGCAGTCAAAGTAA

ATTAAAGCTAACAGTGTCTAGGTGTGTGTTTGAGGCTTGCATGGTAGGCATTATTAGTAA

TGGAACTCTGAGCTGTCGCCACTGCACAGGCCACAACATTTACTGCCTTTGCTACTTAAT

GGGCCCTGGAAAGTTTGTAGGCCTTAATATATTAAATTCAAACAAGTTTTATGAGTCTGG

CCTCACTGAAATGCTTAGCTGCCATGGAAATAAAATCTTGCCTTTAGCCACATTACACAT

TTGCGGCAGTTTTCACCCTTTTCCTGACTTTAAAAACAGCATGCTTACTCGCTGCAAAGT

CTTCGTTGGTGAGCGCATTGGCGTGTTTTCACCTCTGAATGTGAGTCTAAGCTACAGCTC

TGTTGTGGCAGACAAGGGGTCGTTCCAGAGCTTGAACCTCAACTACACTTTCCACCAAAC

CACAACTATTTGGAAGTT

>Seq27 [organism=Adenovirus] [isolate=Goat adenovirus 1-2023-Shannxi]

TCAACAGGCGGCACCAAACAGAGCCAATTATATTGGCTTCAGAGATAACTTCATAGGATT

AATGTATTACAACAGCAACGGTAACCTAGGGGTGTTGGCCGGCCAAGCTTCACAATTAAA

TGCGGTGGTCGATCTACAAGACCGTAACACAGAACTTTCTTATCAGTTGATGCTGGATAA

CTTATATGACAGAAGTCGCTATTTCAGTATGTGGAATCAGGCCATTGATAGCTACGACCC

AGATGTAAGAGTGATTGAAAATCACGGCGTGGAAGATGACATGCCAAATTACTGTTTTCC

ATTAAGTGGGGTAGTGCCAAGAGCTACTTCAGTTCGCGTTCAGCGCAATGACCAGAATGA

TGCCTGGATTGCTGCAGGCAACAACTCCAATAACAATTACATCAACAAAGGAAACTTGGA

AGCTATGGAAATCAATTTGGCCGCTAACCTTTGGAAAGGGTTCCTCTACTCTAACGTGGC

CCTGTACCTGCCAGATGACCTTAAGTTTACGCCACCTAACGTTACTCTCCCAACCAACAC

TAATACGTACGAGTACATGAACGGGCGCCTTCCCGCCAGCGGTCTTATTGATACTTATGT

CAACATCGGGGCTCGGTGGTCGCTGGACGTAATGGATAACGTGAATCCTTTCAACCACCA

CAGAAACTCTGGCCTCCGTTACCGCTCGCAGCTCCTCGGCAATGGGCGGTACTGTCAGTT

TCACATTCAAGTGCCCCAGAAGTTCTTTGCCATCAAGAACTTGCTCCTGCTCCCTGGCAC

GTACACTTATGAATGGTCTTTTAGAAAAGACGTCAACATGGTGCTTCAAAGCACTTTGGG

AAACGATCTCCGCGTGGATGGCGCCTCCATCAATATTGACAGCGTCAACCTCTACGCTAG

CTTCTTCCCAATGGCCCACAATACTGCCTCCACCCTTGAAGCAATGCTCAGAAACGACAC

TAACGATCAATCCTTCATTGATTACCTGTCCTCCGCCAACATGCTCTATCCCATCCCCGC

TAACGCCACCAACCTACCCATCTCCATTCCATCTAGAAACTGGGCCGCTTTTAGAGGTTG

GAGCTTCACCCGCATTAAACAGCGAGAAACTCCAGCTTTGGGTTCTCCTTTTGACCCTTA

CTTTACTTACTCTGGTACTATTCCGTATTTGGATGGCACCTTTTACCTCAACCACACGTT

CCGCCGCGTCTCTATCCAATTTGACTCTTCAGTCCAGTGGCCAGGTAATGACAGACTGCT

CACCCCCAATGAGTTTGAAATAAAGAGAACAGTGGACGGAGAAGGCTATACAGTAGGTCA

GAGTAATATGACTAAGGACTGGTTTTTAGTTCAGATGCTAGCTAATTATAACATTGGCTA

TCAAGGCTACCACCTCCCAGACGGATACAAAGATCGGACGTATTCATTCTTACGCAACTT

CCAGCCCATGTGTCGCCAAGTAGTTGATAATGCTAACTTTGCTTCCTACCAAGACGTTCA

GCTTACCAACCAGCACAATAATTCAGGGTTTGCTGGCTTTGCAAGCGCTGCCCTGTGCAG

GGAAGGGCATCCTTATCCTGCTAACTGGCCTTATCCGCTTATTGGCCCAAATGCCGTCAC

CGCCACTACCACCCAGAGAAAGTTTTTATGTGACCGCACTTTGTGGCGCATACCCTTTTC

TTCCAACTTTATGTCTATGGGCTCGCTCACAGATCTTGGTCAGAATTTACTGTATGCAAA

CGCAGCCCACGCACTTGACATGACTTTTGAAGTGGATGCCATGGAAGAGCCCACATTGCT

CTATGTCCTATTTGAAGTGTTTGACGTTGTGCGCGTACACCAACCCCACAGGGGCGTCAT

CGAAACAGTGTACCTGAGAACCCCCTTCTCCGCGGGTAACG

>Seq28 [organism=Adenovirus] [isolate=Goat adenovirus-2022-Ningxia]

TAATTGGCCTTATCCGCTCATTGGAGAAAACGTAGCCCCATGCACTACCCAGAAAAAGTT

TTTGTGTGACAGGACTCTGTGGCGCATCCCATTCTCTAGCAATTTTATGTCCATGGGCTC

GCTCACTGACTTGGGTCAAAACTTGTTGTACGCTAACGCTGCTCATGCGCTAGACATGAC

TTTTGAAGTGGATGCTATGGATGAACCTACATTGCTGTATGTGCTGTTTGAGGTATTTGA

TGTTGTGCGCGTGCATCAACCACACAGGGGCGTCATTGAAACCGTGTACCTGAGAACTCC

GTTCTCTGCTGGCAACGCCACTACATAATCGCCGCGATGGGCTCCAGGGAAGAAGAGTTG

CGAGCTATTGTTCGCGACTTAGGCATAAGTCCTTACTTTTTAGGAACTTTCGACAAGCGC

TTCCCAGGTTTCTTGCATAGAGATAAACTGAGTTGCGCGATTGTGAATACAGCGGCCCGC

GAGACTGGTGGGGCCCACTGGCTAGCGCTAGCTTGGTTTCCAAATGCTAAAAACTTTTAC

TTTTTTGACCCGTTTGGGTTTTCTGACCATAAACTTAAACAGATTTATCAGTTTGAATAT

GAAGGCTTGTTGCGCCGAAGCGCGCTAGCGGGCGATGGCTGCGTAAATTTAGTGAAAAGC

ACTGAAACAGTTCAAGGTCCAAACAGCGCCGCCTGTGGGTTGTTTTGTTGCATGTTTTTG

CATGCTTTTGTAAATTGGCCAGACCGCCCAATGAACCGCAACCCCACAATGGATTTGCTA

ACTGGTGTACCTAATGCTGATATGATGAAACTGTCGTCGCTTGGGATTTTAAGGGAAAAC

CAAAATCAGCTGTATAAATTTTTGTCATCCCACTCCCCATACTTTCGCGCTCACCGCCCC

CAGATTGAACGCGATACCTCTTTTAATAAACTGCTAGAACTCAAAAATCAATAAACTGAA

CTTTATTGAACTTTTGCGTGTCAGAATTTTGCTTTAAAAAAGACATTCGTCGGCATCATC

TTGGCCGGTGGGGAGGAGAGTGTTTTGGACTCTGTATTGAGGTTGCCACTTGAACTCTTG

AACCACAATGGAAGCTTTAGTGCCAGTGAGCGAAGACCACATTTGCTTAGCCAGCTGCAG

AGCCATCACAACATCAGTGGAGCTTATTTTAAAATCACAATTTTTCTGAGGATTCGCTTT

AGTGTTGCGGAACACTGGGTTGCAGCACTGAAACACGAGAACCACAGGGTTGTTTAAAGT

AGCTAGCACTTTGGCATCTTCCACCAAAGAACGATCGATGTTGCTGACGGCATTCAAAGC

AAAAGGGGTAACTTTGCAAGTTTGCTTGCCAAGAAGAGGAATAACGTGGTGGCCGTAGTT

GCATTCACACACCAACGGCATCAACAGCATCTCGCCGGCCTTGGGCATCTGCGGATACAT

GGCTTTTACAAAATCTCTAATTTGGTAAAAGCCTTGACGGGCTTTGTTCCCGTCAGAATA

AAAGTATCCGCAAGATTCAGAGCTAAAAGAATTTAAAGAAGACTTTAGATCATAGAAGCA

GCACATGGCGTCGGCATTTTTCATCTGCACCACACTGCGACCCCACTTGTTGGTGACAAT

TTTAGCCCTCTCTGGCGACTCTTTTAGAGCGCGTTGGCCGTTCTCG

>Seq29 [organism=Sheep polyomavirus 1] [isolate=Goat polyomavirus-2023-Shannxi]

CAGGAAACTACCTTTACATCTTCAGTTTCCTCAGGTGTAAAATAGTTTTCTCTAAGTC

CCCCAGGTACAGACTCTTGTAACAATGTAAAGGGGGCTTTACAAAGAGCAACATATAATT

CATACTGTTTAATAACTGCTTTAACAATTACAAAACTTATACTGCAATACTTATTACAGA

AATTATAGATAGCACTGACTCTATGCTTATTGGGCGTTAACAGATATAACAGAGTATTAG

TAGTATATCCATGTCTTGAGATGAAACTGCAACAGAACTTATCCAGTAGTTTCTTATATA

ACAAACCCCCCTTAGAATGAGTAGTATACACAATAAAGCAGTTAACAGTTTTGTTACTAA

ATACAGCCTTACTAAGCATATCTTGAATTTCAGCAGGTAAATCCTGAGGGTGGGTCTGTC

CTTTATCCTTAGGTGGTGTGGACTGAGATCCCGGGATGTCCTCTTCTGTCCTGGCTCTTT

TGGGGGTGGGCTCCTCCTCCTCCATTTCTTCAGGACAAAACAGGTCTGCATTGAAGGCCT

CCCACCAACTCTCCCATTCTGGGGTTCCATAAGGTGGGACCTACAAAAAGAGCATTTAGA

TAAATAGAGATTTAACATCCCTCCCAGGCAACTATACTTACTTAGAGATTCAGATGTCTA

AATGGAGTGTCTGCCAGAATGGACACCCAAAGCTGGGCAGTGGAGTATTCTCTTGGCAAG

CCAAACCAGGTGAGAAAGCAGTCATAGCAGTAGCATCTCAGCCATACATTAGGACGCCAA

CAGACATTTCCCTTGCTCCTCAGATGGCTACGGTGCAGCTGACACATTAAGCAGGTGCAG

GAAGCCACTCTATAAGAGGCACACAAATTCCAGTCCTTAATATACATAGAGTCCACCTCA

CCCACCTCTTGTGGAGACCAGCTATAATTCCAGAACTCCTCTTCCTGAGACTCTTTTATG

TTAGCCTCCATGGTTCTATACAGAGCATTCAATCTTTTCATCTGGTTCTCATCACCACCT

TTATCTGGATGCATTTCTTTGCATTTAGATAAAA

>Seq30 [organism=Sheep polyomavirus 1] [isolate=Goat polyomavirus-2022-Ningxia]

GCTTTTACTTCTTCCCCTTCTTCGGGGGAAAAATAATTCTCACGCAGGCCTCCAGGTACT

GACTCCTGCACAAGCGCAAAAGGGTCTTTACACAGAGCAGAATACATTTCAAATTGCTTA

ATAACAGCTTTAACAATTACAAAGCTAACACTACAATACTTGCTTGCGTAATTCTGCACA

GCACTAACCCGGTGCTTATTTGGAGTTAAAAGGTATACAATATTATTGGATACATAAGAG

TGTCTGCTTATAAAGGTACAGCAAAATTTATCCATTAATTTGTTATAAAGCAGTGGGCCC

TTCCCCTCTGTGGTATACACTACAAAGCAGGTTATACATTTATTACTGAAAATTGCCTTA

CTAAGAAAGTCCTGCAAGCATTCTGGAAGATCTTTAGGATGCTCTGGTTTCTGCTTTGGG

GGGGTTGATTGAGATCCCGGCATTTCATCTCCTCTACTTTTCTTAGGTGGTGGCTCTTCA

AATTCAGGCATTTCTTCAGAGCAGAAGAGGTCCTCATTGAAGGTATCCCACCATCTCTCC

CACTCTGGGGTTCCATACTGGGGCACCTGAAATATAAGATGATGAGAGCATGGAAGTACT

TGGGGACATTCCAATGAAGAACTTGCACTTACTTTATTAAAGGTTTAGGTTCTTCATTGG

TGTCTGCCCAATGACTTCACACCACACTTTGGCTGTTTCGTAGTCCGTGGGAAGTCCAAA

CCACTCACAGAAACAGGCAAAGCAGTAGCATCTCAGCCATACATTAGGACGCCAGCATAC

ATTTCCTTTGCTTTGCTGATGACTCCTTGCTAGGCGGCACATTAGACAGCAGCAGTTAGC

TACTCTGTTAGTTGAGCAAAGCACCCACTCTCTAATGTAGAGAGTGTCTGGAGGCTCATC

ACCCACCTCTTCAGGTGTCCAGGAGTATCTCCAAAATTCTGCATCCTGCACTTCTTTGAT

ATTAGCTTCCATGGTTCTGTAAAGGGTATTCAGCTTCTTCATATTTTCCTCATCCCCACC

TTTATCTGG

>Seq31 [organism=Herpesvirus] [isolate=Goat herpesvirus-2022-Ningxia]

AAATCACCCTGTGGTCCTCCCCGGCCATGCGAAATCTGGCCAACGGGCTCAGCAACATGG

GCTACCAGGTGTTTGAGGCCAACGTGGATGCCTCCACTCGGTTCATCATCGATAACCAGT

TTTCTACCTTCGGCTGGTACACGTGCAGCAACTTTTCTGCCCGAGCGAGTGACCACCGCG

ACTCCCACACGCAACTGGAGTACGACTGTGCCGTGGGTGATGTGCGCTTCGACCCTCACC

GCCTGGACTGGCCCCACTACCACATCCTATCCTTTGATATAGAGTGCCTGGGCGAGTGCG

GCTTCCCCACGGCCGATAGGGACGAGGACATCATTATCCAGATATCCTGCGCTATCTGGA

CCGTGGGCTGCGAGAGCGGGCCTAAACTTATTCTGCTTTGCGTGGGCACCTGCGCCCCCC

TGGACGGTATAGACGTGTACGAGTTTCCCTCTGAGATGGACCTGCTGTATGACTTTTTCA

CCCTGATCAGGGACTACGGCGTGGAGATAATCACGGGCTACAATATCTGCAATTTTGACT

TCCCCTACGTGCTGGATAGGGCTCGTCACGTGTACAACCTCAAACCCGAGGAGTATAGCA

AGACAAAGACCAACTCTCTTTTTTACGTGTACAAGCCCCAGGAGGGAAACTTTATGCGGG

CGCATTCTAAGGTCAAGCTATCCGGGGTGGTAGTCATTGACATGTATCACATCTGCCGGG

AAAAGCTAAGCCTCTCTAACTATAAACTGAACACAGTGGCCAAGGAATGCATGGGCGAGG

CCAAGACAGACGTCACTTACAAAGACATTCCCATCCTATTCAGGGGCTCCAGCCACGACC

GGGCCAAACTGGGCCTGTACTGCGTACAGGATGCTGTCCTGGTCCTGAACCTCCTGCAGC

ACTTTATGACTCACATTGAAATCACGGAAATTGCTAAAATTGCCAACATCCCCCCCAGAC

GAGTCATTACTGACGGGCAGCAGATTAGGGTG
